# Applying DNA Barcodes to Identify Closely Related Species of Ferns: A Case Study of the Chinese *Adiantum* (Pteridaceae)

**DOI:** 10.1371/journal.pone.0160611

**Published:** 2016-09-07

**Authors:** Fan-Hong Wang, Jin-Mei Lu, Jun Wen, Atsushi Ebihara, De-Zhu Li

**Affiliations:** 1 Germplasm Bank of Wild Species, Kunming Institute of Botany, Chinese Academy of Sciences, Kunming, Yunnan, China; 2 College of Life Sciences, University of Chinese Academy of Sciences, Beijing, China; 3 Department of Botany, National Museum of Natural History, Smithsonian Institution, Washington DC, United States of America; 4 Department of Botany, National Museum of Nature and Science, Tsukuba-shi, Ibaraki, Japan; Chinese Academy of Medical Sciences and Peking Union Medical College, CHINA

## Abstract

DNA barcoding is a fast-developing technique to identify species by using short and standard DNA sequences. Universal selection of DNA barcodes in ferns remains unresolved. In this study, five plastid regions (*rbcL*, *matK*, *trnH-psbA*, *trnL-F* and *rps4-trnS*) and eight nuclear regions (ITS, *pgiC*, *gapC*, *LEAFY*, ITS2, *IBR*3_2, *DET*1, and *SQD*1_1) were screened and evaluated in the fern genus *Adiantum* from China and neighboring areas. Due to low primer universality (*matK*) and/or the existence of multiple copies (ITS), the commonly used barcodes *matK* and ITS were not appropriate for *Adiantum*. The PCR amplification rate was extremely low in all nuclear genes except for *IBR*3_2. *rbcL* had the highest PCR amplification rate (94.33%) and sequencing success rate (90.78%), while *trnH-psbA* had the highest species identification rate (75%). With the consideration of discriminatory power, cost-efficiency and effort, the two-barcode combination of *rbcL*+ *trnH-psbA* seems to be the best choice for barcoding *Adiantum*, and perhaps basal polypod ferns in general. The nuclear *IBR*3_2 showed 100% PCR amplification success rate in *Adiantum*, however, it seemed that only diploid species could acquire clean sequences without cloning. With cloning, *IBR*3_2 can successfully distinguish cryptic species and hybrid species from their related species. Because hybridization and allopolyploidy are common in ferns, we argue for including a selected group of nuclear loci as barcodes, especially via the next-generation sequencing, as it is much more efficient to obtain single-copy nuclear loci without the cloning procedure.

## Introduction

DNA barcording is a method to achieve accurate and rapid species identification by using short and standard DNA regions [1]. To find a locus that is universal, readily sequenced and has sufficiently high sequence divergence at the species-level, Chase *et al*. [2] assessed *rbcL* and nuclear ITS, and found both markers performed well in identifying plants. Kress *et al*. [3] initially proposed *rbcL* as a DNA barcode for plants because of its high universality. A global plant DNA barcode system was evaluated by comparing amplification universality and sequence divergence levels for nine putative barcode loci [4], and they recommended the combination of *rbcL*+ *trnH-psbA* as a two-locus global land plant barcode. Hollingsworth *et al*. [5] evaluated seven candidate plastid regions (*rpo*C1, *rpo*B, *rbcL*, *matK*, *trnH-psbA*, *atp*F*-atp*H, and *psb*K*-psb*I) in three divergent plants groups. Their results revealed that no single locus had high levels of universality and resolvability in these groups [5], and they proposed various three-locus combinations involving *rpo*C1, *rbc*L, *matK* and *trnH-psbA* to identify these groups. CBOL plant working group [6] recommended a two-locus combination of *rbcL*+ *matK* as the land plant barcode. The second internal transcribed spacer (ITS2) was proposed as a universal DNA barcode [7], and then the China Plant BOL Group further argued that ITS/ITS2 should be incorporated as a core barcode for seed plants [8]. In the past five years, DNA barcoding is fast evolving to include genome skimming [9].

DNA barcoding studies on ferns (monilophytes) and lycophytes are relatively few in comparison with those on seed plants, even though DNA barcoding may be of great value on the identification of their gametophytes, a free-living and featureless generation in the life cycle. Ebihara *et al*. [10] tested the utility of *rbcL* and *trnH-psbA* using 733 taxa, and demonstrated that these two barcodes were effective to identify the Japanese pteridophyte flora. de Groot *et al*. [11] evaluated the discriminatory power of *rbcL* and *trnL-F*, and suggested *rbcL* + *trnL-F* can be used as a two-locus barcode to identify NW-European fern species. Li *et al*. [12] assembled sequences of *rbcL*, *matK* (designed specific primers for each of the major clades), and *trnH-psbA* from 74 species of 37 families, and *trnL-F* from 32 species of 19 families in major fern lineages. They suggested that *matK* + *rbcL* can provide a two-locus barcode with strong resolving power in ferns, and the study favored *trnL-F* over *trnH-psbA* as a potential back-up locus if the universal primers of *matK* failed. Schneider & Schuettpelz [13] used *rbcL* sequence to determine the identity of a sterile gametophyte of unknown origin, and successfully identified it as *Osmunda regalis*. Li *et al*. [14] developed a procedure “Tissue-direct PCR”, which can make the identification of diminutive and characterless stages of ferns (gametophytes and young sporophytes) easy and rapid when it was combined with plant barcodes. Pryer *et al*. [15] used *rbcL*, *atpA*, and *trnG-R* sequences to identify a cultivated plant marketed as *Cheilanthes wrightii* in the horticultural trade but the plant was actually *C*. *distans*.

At present DNA barcoding in ferns has relied solely on plastid loci, which are uniparentally inherited [10–13,15]. Ferns are characterized by frequent hybridizations among closely related species [16] as well as polyploidy [17–18], and have relatively frequent apomictic lineages [19]. DNA barcoding was thought to likely show low levels of discrimination success rate in taxa having high rate of hybridization and polyploidy [20]. Combining biparentally inherited nuclear barcodes with uniparentally inherited plastid barcodes may be useful for species discrimination in allopolyploid species and those of hybrid origin.

The Chinese *Adiantum* is herein recognized as a good model to evaluate the candidate barcodes because most species in the region have been clearly defined, while a few problematic species can be used to test the effectiveness of selected barcodes. *Adiantum* consists of about 150~200 species, of which most species are distributed in the tropical to subtropical regions, with the greatest diversity in the Neotropics [21–24]. Ching [25] treated *Adiantum* as an early diverged and unique genus, and recognized the monotypic family Adiantaceae. Smith *et al*. [26] included *Adiantum* in Pteridaceae and Tryon *et al*. [24] recognized the group as a subfamily Adiantoideae in Pteridaceae. Molecular evidence supported the placement of *Adiantum* in Pteridaceae [27–29]. There are some eurychoric species showing high morphological divergences due to divergent habitats, while there are also high morphological similarities among some species, especially in series *Venusta* [23,30]. Lin [23] divided Chinese *Adiantum* into seven series: *Reniformia*, *Gravesiana*, *Caudata*, *Pedata*, *Flabellulata*, *Venusta*, and *Venericapilliformia*, and recognized 31 species, five varieties, and four forms in China. Lin *et al*. [30] recognized three additional species, *A*. *meishanianum* F. S. Hsu ex Yea C. Liu & W. L. Chiou and *A*. *formosanum* Tagawa from Taiwan, and *A*. *subpedatum* Ching from Zhejiang province of eastern China, with a total of 34 species and three varieties of *Adiantum*, of which 16 are endemic to China.

The DNA barcoding approach has been greatly advocated since the concept was proposed [1], and it has been shown to be an important tool for species identifications, and a supplement to traditional morphology-based taxonomy [31–32]. Combining DNA sequences with existing morphological characters may facilitate species identification and classification [33–36].

The objectives of this study are to (1) screen and evaluate potential plastid and nuclear barcodes in *Adiantum*; (2) combine the barcodes with morphological characters for assessing species delimitations in several species groups of *Adiantum*; and (3) discuss general guidelines for barcoding fern species.

## Materials and Methods

### Taxon Sampling

A total of 154 samples representing 33 species of *Adiantum* were collected in this study. The taxon names were mainly based on the recent treatments [30,37]. Six samples of *A*. *menglianense* [38] and two samples of *A*. *ailaoshanense* were also included in this study. At least two individuals were sampled from different populations of each species except for the stenochoric species *A*. *fengianum*, *A*. *mariesii* and *A*. *lianxianense*, which were only sampled from one population. More individuals (3~20) of eurychoric species were sampled to represent their distributional range. All taxa included in this study, together with voucher information, were listed in S1 and S2 Tables (Supporting information).

### DNA extraction, PCR amplification and sequencing

Total DNAs were extracted from silica-gel dried-leaf material and herbarium specimens using the CTAB procedure [39] and Dneasy (QIAGEN) extraction kits. Polymerase chain reaction (PCR) amplifications were performed in a 20 μL reaction mixture containing 1×Taq buffer [50 mM (NH_4_) _2_SO_4_; 75 mM Tris–HCl (pH 8.3); 50 mM KCl; 0.001% gelatin]; 2.5 mM MgCl_2_, 0.4 mM of dNTPs, 0.5 μM of each primer, 1.0 U of Taq DNA Polymerase (TaKaRa Biotechnology Co. Ltd., Dalian, China), and 1 μL of genomic DNA (25–30 ng). Purified PCR products were sequenced in both directions with the PCR primers on an ABI 3730xl DNA Sequencer (Applied Biosystems, Foster City, USA).

Five candidate plastid regions (*rbcL*, *matK*, *trnH-psbA*, *trnL-F* and *rps4-trnS*) and eight candidate nuclear regions (ITS, *pgi*C, *gap*C, *LEAFY*, ITS2, *IBR*3_2, *DET*1, and *SQD*1_1) [40–48] were screened and evaluated in *Adiantum*. The primer information and thermocycling conditions used in this study were listed in S3 Table.

A subset of samples were sequenced via direct sequencing without cloning using the amplification primers of *IBR*3_2, but this resulted in chromatograms with multiple peaks for most species. We then cloned *IBR*3_2 from all samples, using the ZeroBack Fast Ligation Kit (Tiangen, Beijing) and following standard protocols for cloning, colony selection, and post-cloning re-amplification with pZeroBack/Blunt Vector primers. At least six and up to 20 colonies were picked for sequencing for each individual. Some taxa ultimately yielded fewer than six sequences despite multiple cloning attempts because of technical difficulties in generating sequences.

### Data analysis

Sequences were aligned with Clustal X [49] and then manually adjusted in Geneious 4.8.2 (Biomatters Ltd., NZ). The genetic pairwise distance for each marker was calculated using MEGA 5.2 based on pairwise deletion and the *P*-distance model [50]. Intra- and inter-specific genetic divergences of the four candidate DNA regions were analyzed by Wilcoxon signed-rank tests [51], and “barcoding gap” was estimated by comparing the intra- and inter-specific divergences of each candidate locus using taxonDNA [52]. The neighbor-joining (NJ) trees were constructed based on single markers and combinations of two/three/four markers in MEGA 5.2, with pairwise deletion based on the *P*-distance model, and used to evaluate whether individual samples of a species clustered in species-specific monophyletic clades. Robustness of inference was assessed by running 5, 000 bootstrap replicates [53].

## Results

### Screening the DNA barcodes for *Adiantum*

For the plastid DNA barcodes, *matK* showed the lowest PCR amplification success rate (33.33%), while *rbcL*, *trnH-psbA*, *trnL-F*, and *rps4-trnS* showed much higher PCR amplification and sequencing success rates. PCR amplification success rate of the four DNA regions was 94.33% (*rbcL*), 93.20% (*trnH-psbA*), 85.82% (*trnL-F*), and 94.20% (*rps4-trnS*), and sequencing success rate of the four markers was 90.78%, 84.16%, 77.01%, and 83.57%, for *rbcL*, *trnH-psbA*, *trnL-F*, and *rps4-trnS*, respectively (Table 1). Thus, these four barcodes were used for subsequent analyses in this study.

**Table 1:** Summary of genetic variability and sequence characteristics of the candidate barcodes and their main combinations in this study.

	*rbcL*	*trnH-psbA*	*trnL-F*	*rps4-trnS*	R+S	R+P	R+F	P+S	P+F	F+S	R+P+S	R+P+F	R+F+S	P+F+S	R+P+F+S
No. taxa	30	28	30	30	29	28	29	27	27	29	27	27	27	25	24
No. sequences	133	122	116	126	110	113	108	104	93	99	95	89	92	82	77
Aligned length (bp)	1143	520	826	792	1935	1663	1969	1312	1346	1618	2455	2489	2761	2183	3281
Average intra-distance (%)	0.06	0.05	0.16	0.10	0.06	0.06	0.09	0.10	0.13	0.14	0.07	0.09	0.10	0.13	0.10
Average inter-distance (%)	5.74	7.38	15.76	10.69	7.62	6.22	9.38	9.62	12.68	13.04	7.70	9.16	9.85	12.14	9.72
Variable sites (%)	23.10	28.85	53.87	42.55	30.85	24.23	35.65	36.51	43.09	47.28	29.61	33.19	36.98	40.95	34.75
Informative sites (%)	21.96	27.88	52.30	40.40	29.20	23.03	34.33	34.68	41.60	45.80	27.94	31.90	35.46	38.75	32.64
PCR success (%)	94.33	93.20	85.82	94.20	N/a	N/a	N/a	N/a	N/a	N/a	N/a	N/a	N/a	N/a	N/a
Sequencing success (%)	90.78	84.16	77.01	83.57	N/a	N/a	N/a	N/a	N/a	N/a	N/a	N/a	N/a	N/a	N/a
Identification success (%)	73.33	75	66.67	77.33	79.31	78.57	79.31	81.48	77.78	79.31	77.78	77.78	92.59	92.00	92.00
No. unidentified species†	2	1	2	2	2	0	0	1	0	0	0	0	0	0	0

† the number of unidentified species except for four species groups.

For the eight nuclear DNA barcodes, the PCR amplification rates in seven candidate nuclear regions (ITS, ITS2, *pgi*C, *gap*C, *LEAFY*, *DET*1, and *SQD*1_1) were low (<50%) or did not obtain clean sequences by direct sequencing without cloning. *IBR*3_2 had 100% PCR amplification success (S1 Fig), but only 23 sequences of 13 species were obtained by direct sequencing, and additional 244 sequences representing 53 individuals of 32 species (including a recently published species *A*. *ailaoshanense*) were obtained by sequencing the clones (S2 Table). *IBR*3_2 had sufficient discriminatory power in series *Caudata*, and was used to identify hybrid individuals in *Adiantum* in the present study.

### Variation of barcoding markers

The newly acquired DNA sequences have been deposited in GenBank and their accession numbers were provided in S1 and S2 Tables. Fourteen sequences (*rbcL*: 5; *trnH-psbA*: 1; *trnL-F*: 4; *rps4-trnS*: 4) were downloaded from GenBank. The data analyses included 133 *rbcL* sequences, 122 *trnH-psbA* sequences, 126 *rps4-trnS* sequences, and 116 *trnL-F* sequences.

Homopolymer (*e*.*g*., poly-A/G/C) within *trnH-psbA* was detected in taxa in ser. *Reniformia*, ser. *Gravesiana*, ser. *Caudata*, and ser. *Venusta*. The mononucleotide repeats near the end of sequences led to unclean reverse sequences in some cases. So only forward sequences of *psb*A in some individuals were used for the analyses. There are some poly structures in *trnL-F* and *rps4-trnS*, so the sequences of the “f” end of *trnL-F* and those of the *trnS-F* end of *rps4-trnS* of some samples were difficult to obtain. The forward reads alone were added to the analyses in these samples that the mononucleotide repeats exists.

**Fig 1:**
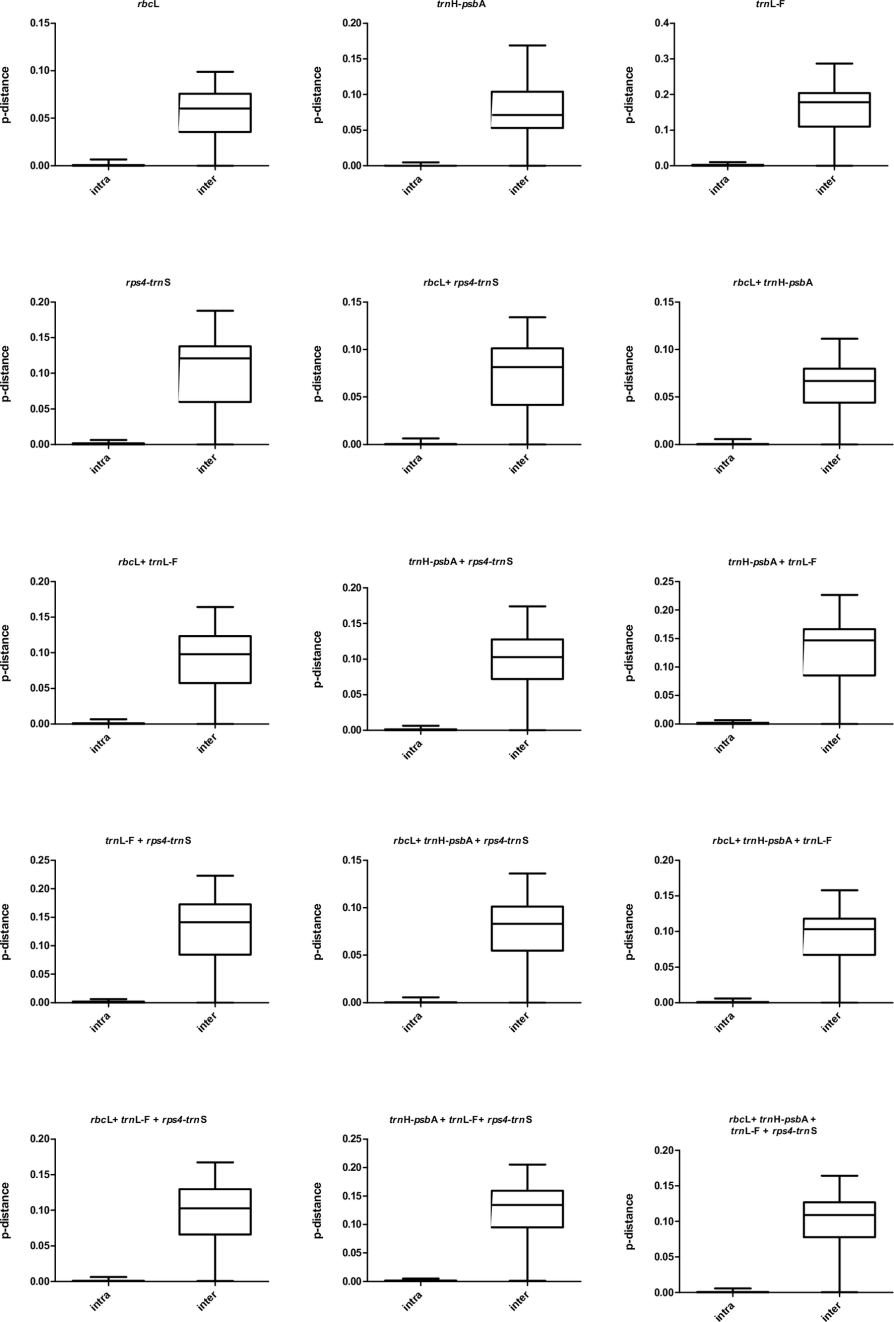
The distributions of divergences for four markers (*rbcL*, *trnH-psbA*, *trnL-F*, and *rps*4*-trnS*).

The *trnL-F* marker had the highest mean intraspecific divergence followed by *rps4-trnS*, *rbcL*, and *trnH-psbA*, and had the highest mean interspecific divergences followed by *rps4-trnS*, *trnH-psbA*, and *rbcL* (Tables 1 and 2). The interspecific and intraspecific genetic divergences of the four DNA regions were analyzed with the Wilcoxon signed-rank tests [51]. At the intraspecific level, genetic divergences exhibited no significant difference among the four barcodes (Table 3). The barcoding gap was detected for the four markers, which was indicative of high sequence variation among species for the four barcodes (Fig 1).

**Table 2:** Wilcoxon signed-rank tests of interspecific divergence among DNA markers.

W+	W-	Relative ranks		N-value	P-value	Result
		W+	W-			
*trnH-psbA*	*rbcL*	44905	7745	325	≤0.001	*trnH-psbA* > *rbcL*
*trnL-F*	*rbcL*	61424	1	351	≤0.001	*trnL-F* > *rbcL*
*rps4-trnS*	*rbcL*	70933	320	378	≤0.001	*rps4-trnS* > *rbcL*
*trnL-F*	*trnH-psbA*	44832	18	300	≤0.001	*trn*L-F > *trnH-psbA*
*rps4-trnS*	*trnH-psbA*	52279	371	325	≤0.001	*rps4-trnS* > *trnH-psbA*
*rps4-trnS*	*trnL-F*	14	61411	351	≤0.001	*rps4-trnS*<*trnL-F*

**Table 3:** Wilcoxon signed-rank tests of intraspecific divergences among DNA markers.

W+	W-	Relative ranks		N-value	P-value	Result
		W+	W-			
*trnH-psbA*	*rbcL*	39	66	24	0.397	*trnH-psbA = rbcL*
*trnL-F*	*rbcL*	147	24	24	0.007	*trnL-F = rbcL*
*rps4-trnS*	*rbcL*	90	46	26	0.255	*rps4-trnS = rbcL*
*trnL-F*	*trnH-psbA*	98	7	22	0.004	*trnL-F = trnH-psbA*
*rps4-trnS*	*trnH-psbA*	85	20	24	0.041	*rps4-trnS = trnH-psbA*
*rps4-trnS*	*trnL-F*	40	150	24	0.027	*rps4-trnS = trnL-F*

### Applicability for species discrimination

A tree-based method (NJ) was used for the species identification of *Adiantum*. Based on the single barcode, *trnH-psbA* showed the highest species discrimination power among the four DNA regions at 75%, followed by *rps4-trnS* (73.33%) and *rbcL* (73.33%), and *trnL-F* (65.52%) (Table 1).

**Fig 2:**
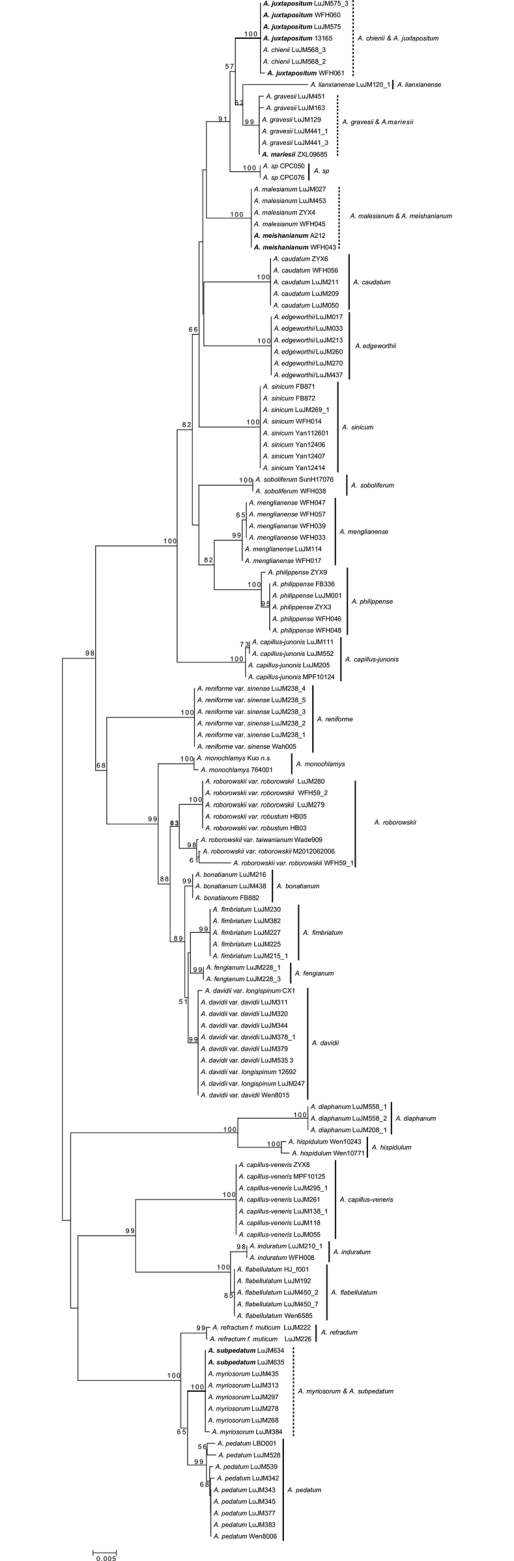
The NJ tree based on the single barcode *rbcL* using the *p*-distance model (dotted vertical line: unidentified group).

**Fig 3:**
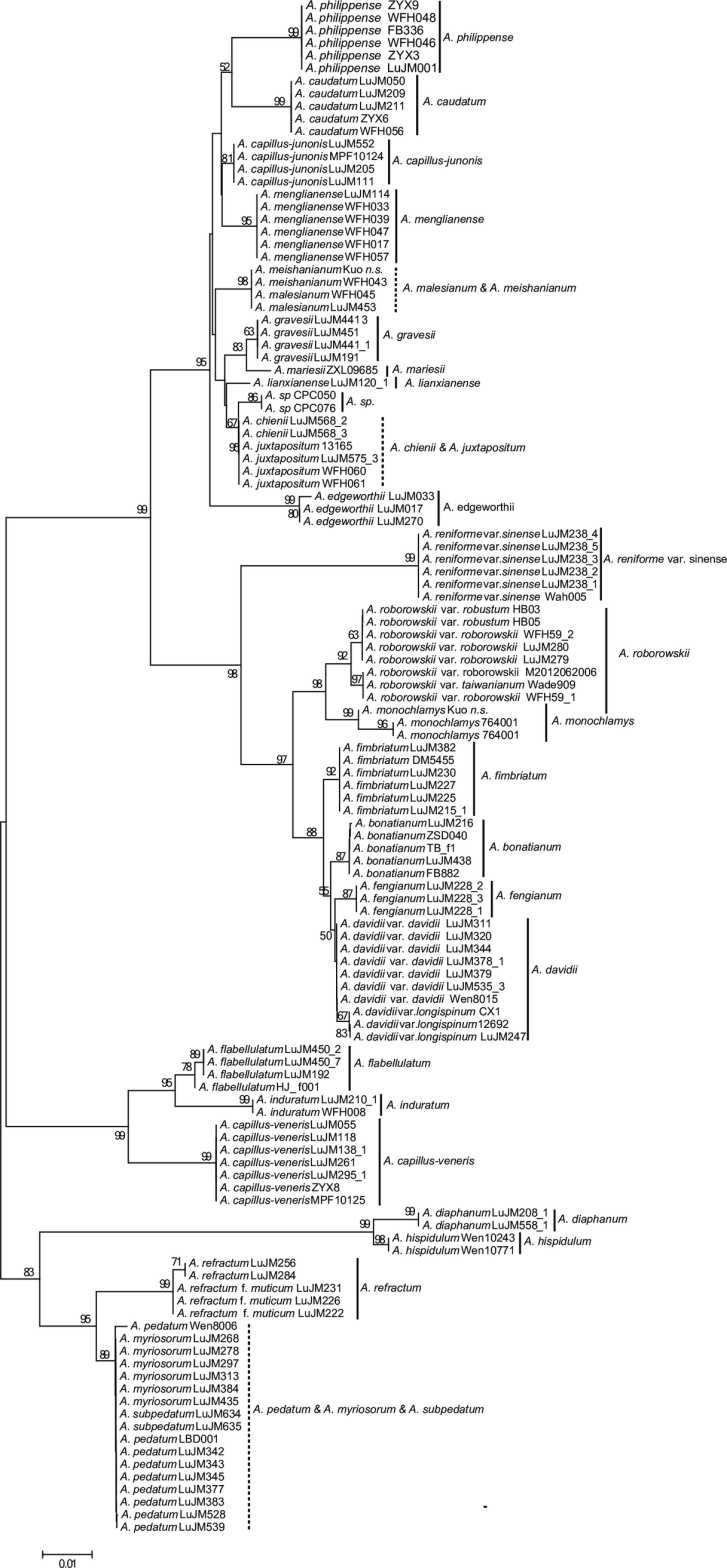
The NJ tree based on the single barcode *trnH-psbA* using the *p*-distance model (dotted vertical line: unidentified group).

**Fig 4:**
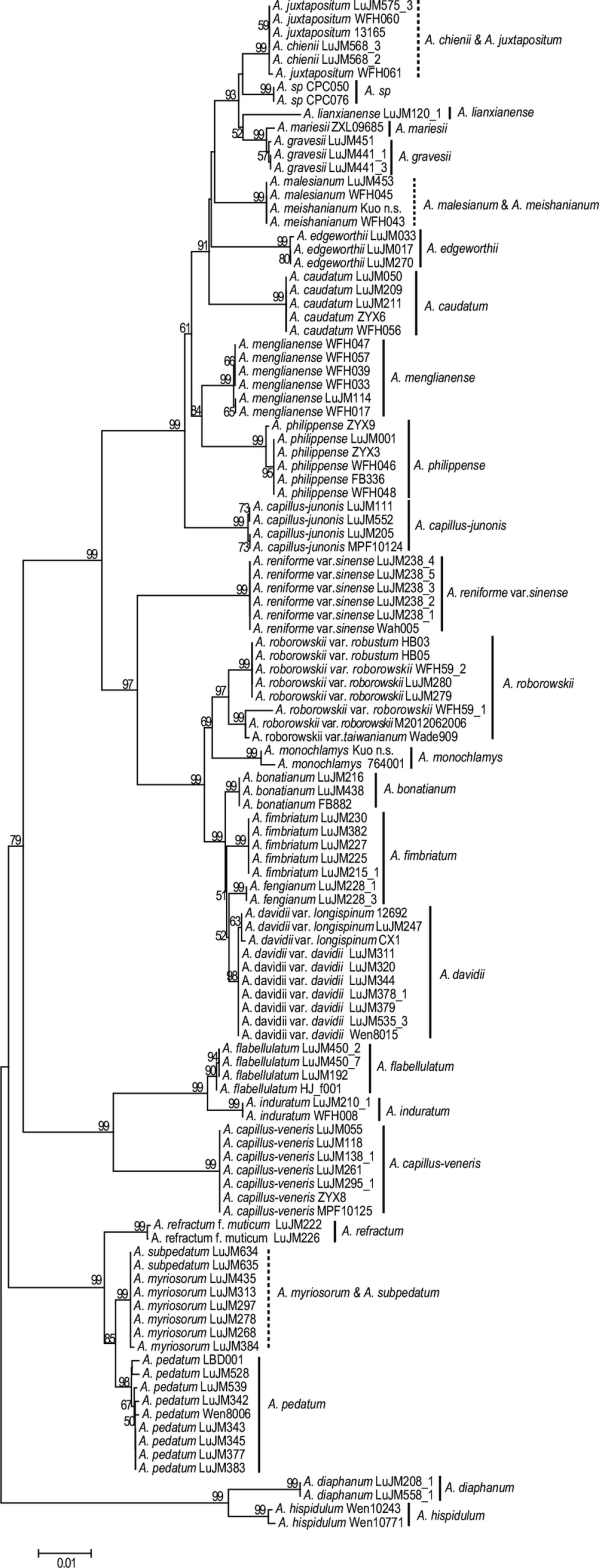
The NJ tree based on the single barcode *rbcL*+*trnH-psbA* using the *p*-distance model (dotted vertical line: unidentified group).

**Fig 5:**
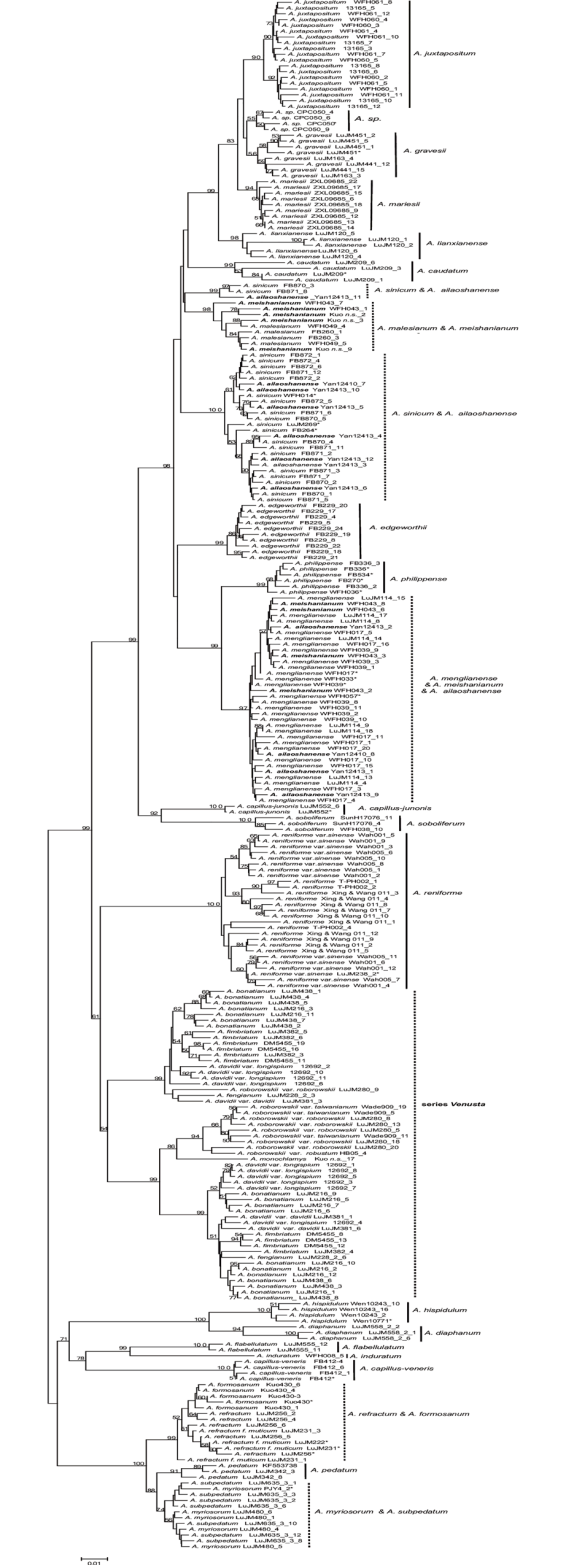
The NJ tree based on the *IBR*3_2 marker using the p-distance model (dotted vertical line: unidentified group).

Four groups of species including *A*. *subpedatum* and *A*. *myriosorum*, *A*. *formosanum* and *A*. *refractum*, *A*. *juxtapositum* and *A*. *chienii*, *A*. *meishanianum* and *A*. *malesianum* were not identified by the present data. *Adiantum formosanum* (Kuo430) strongly clustered with *A*. *refractum*. Samples of *A*. *juxtapositum* (LuJM575, WFH060, WFH 061, CSH 13165) and *A*. *chienii* (LuJM568) grouped together with a high BP value. The samples of two Vietnamese individuals of “*A*. *juxtapositum*” (CP050 and CP076) did not cluster with the Chinese *A*. *juxtapositum* clade (Figs 2–5). Two samples of *A*. *erythrochlamys* Diels (HB03 and HB05 from Hunan) strongly clustered with *A*. *roborowskii* var. *roborowskii* in the NJ trees (Figs 2–5).

Twenty-one of the 28 species were each supported to be monophyletic in the *trn*H*-psb*A tree (Fig 3). Three species—*A*. *davidii*, *A*. *pedatum* and *A*. *myriosorum* were not identified successfully in the NJ tree. However, *A*. *myriosorum* can be distinguished from *A*. *pedatum* by a 9-bp insertion “TTGAAAAGA” in the *trnH-psbA* sequences, and these two species thus can be successfully identified by *trnH-psbA*. *Adiantum davidii* var. *longispinum* fell into a monophyletic group in the *A*. *davidi* clade, even though the *A*. *davidi* clade was only weakly supported, and failed to be identified (BS<50). With the lack of sampling of the close relative *A*. *formosanum*, *A*. *refractum* was successfully identified in the *trnH-psbA* tree.

For *rbcL* and *rps4-trnS*, 22 of the 30 species each formed a clade with high bootstrap values (Fig 3 and S2 Fig). Except for the four unidentified groups described above, *A*. *gravesii* and *A*. *mariesii* failed to be identified in the *rbcL* and *rps*4*-trnS* trees. *Adiantum refractum* was successfully identified in the *rbcL* tree, and *A*. *myriosorum* was successfully identified in *rps4-trnS* tree, with the absence of *A*. *formosanum* and *A*. *subpedatum* in the individual datasets.

Based on the *trnL-F* sequences, 20 of the 30 species were each strongly supported as a monophyletic group (S3 Fig). Five groups of species including *A*. *subpedatum* and *A*. *myriosorum*, *A*. *formosanum* and *A*. *refractum*, *A*. *juxtapositum* and *A*. *chienii*, *A*. *meishanianum* and *A*. *malesianum*, *A*. *gravesii* and *A*. *mariesii* were not identified in the *trnL-F* tree (S3 Fig). *Adiantum mariesii* clustered within the *A*. *gravesii* clade, although the two species differed in two nucleotide positions. *Adiantum davidii* var. *longispinum* fell into a monophyletic group in the *A*. *davidi* clade, although the support value was moderate (BS = 67).

The combination of DNA barcodes can slightly improve the ability of species identification (Table 1).

*IBR*3_2 was successful in identifying the parental species of the presumed hybrid taxa. The maternal parent of *A*. *meishanianum* was shown to be *A*. *malesianum*, and the maternal parent of the hybrid species *A*. *ailaoshanense* was identified to be *A*. *sinicum* in the present analysis (Fig 5). However the interspecific relationship in series *Venusta* was more complicated than other series in *Adiantum* because of the high frequent polyploidy (*e*.*g*., hexaploid of *A*. *bonatianum*, and octaploid of *A*. *davidii*).

## Discussion

### Evaluation of the potential chloroplast barcodes for *Adiantum*

An ideal DNA barcode should be routinely retrievable with a single primer pair with little requirement for manual editing of sequence traces, can provide maximal discrimination among species [6,54], and exhibit a “barcode gap” between intraspecific and interspecific divergences [51].

Although *matK* is one of the most variable coding regions within cpDNA [55–56], it is often difficult to be amplified in ferns because of the loss of the flanking *trn*K exons [57]. Taxon specific primers need to be designed for ferns [12,57]. Even though Li *et al*. [12] endorsed *matK* as a barcode for ferns, *matK* showed low universality in *Adiantum* using the same primers FWPt*matK*F1 and FWPt*matK* rAGK designed by Li *et al*. [12] based on *Cheilanthes* of the same family (Pteridaceae), and primers FWPt*matK* fEDR and FWPt*matK* rAGK designed by Kuo *et al*. [57]. Our study illustrated the need for further *matK* primer development in ferns to ensure efficient PCR and sequencing, and at present this marker seems to be a difficult barcode locus for ferns.

Relatively well-defined gaps between intraspecific and interspecific divergences of the four selected barcodes (*rbcL*, *trnH-psbA*, *trnL-F*, and *rps4-trnS*) were shown in this study (Fig 1). *rbcL* is widely used for molecular phylogenetic inferences and has been proposed as a DNA barcode in ferns as well [10–11,13–14]. Our study also showed that *rbcL* provided the highest level of universality in PCR and sequencing with the primer pair 1F and 1379R, and the second highest species discriminatory power (73.33%).

*trnH-psbA* has been widely used as a plant barcode [3–4,58]. Previous studies suggested that the variation in the relatively short *trnH-psbA* region was enough to differentiate species [10,59]. The species discrimination of *trnH-psbA* was 75% in our study; and only *Adiantum davidii* could not be distinguished (BS<50), except that the four species groups (eight species) cannot be differentiated by all four barcode markers. *trnH-psbA* was ranked the second for PCR amplification success in *Adiantum*, however, its sequencing success rate was lower because of the existence of poly-A/G/C repeats [10,59–60]. *trnH-psbA* can be used as an effective barcode in *Adiantum* with its short length and relatively high variability, and may be used to identify specimens or traditional medicinal materials.

Reported as the most variable locus in ferns [12,61], *trnL-F* is shown here to have the highest sequence divergence, even at the intraspecific level in *Adiantum*. The amplification of *trnL-F* was, however, relatively difficult. PCR amplification success rate was 82.8% when total DNAs were extracted using the CTAB procedure. Higher-quality DNA can improve this rate, and it can reach about 90% when Dneasy extraction kits were used to extract the total DNAs. The sequencing of this marker was also relatively difficult (sequencing success rate at 77.01%) because of the universal presence of mononucleotide repeats in the intergenic spacer. The presence of mononucleotide repeat in *trnL-F* affected sequence quality, and reduced the universality of this marker.

With the rates of sequence divergence just lower than *trnL-F*, *rps4-trnS* ranked the third for PCR amplification rate, but the lowest sequencing success rate. Only a single forward sequence was obtained from 26% samples because of the presence of the mononucleotide repeat structure in *trnL-F* IGS and *rps4-trnS* IGS.

Five species groups including *A*. *subpedatum* and *A*. *myriosorum*, *A*. *formosanum* and *A*. *refractum*, *A*. *juxtapositum* and *A*. *chienii*, *A*. *meishanianum* and *A*. *malesianum*, *A*. *gravesii* and *A*. *mariesii* cannot be identified in the *trnL-F* tree because the species pairs had identical *trnL-F* sequences. *Adiantum refractum* was successfully identified in the *rbcL* tree, and *A*. *myriosorum* was successfully identified in *rps4-trnS* tree, wih the absence of *A*. *formosanum* and *A*. *subpedatum* in the individual datasets. In fact, the identified species were the same by *rbcL*, *trnL-F*,and *rps4-trnS*, with all five groups of species not identified while *trnH-psbA* could identify *A*. *mariesii* from *A*. *gravesii*.

Combination of DNA barcodes slightly improved the ability for species identifications. All combinations except for the three combinations discussed below identified all species except for the four species groups mentioned above. *Adiantum davidii* fell into two respective clades (*A*. *davidii* var. *davidii* and *A*. *davidii* var. *longispimum* in the *trnH-psbA*+*rps4-trnS* NJ tree (S7 Fig). The combination of *rbcL* +*rps4-trnS* failed to separate *A*. *gravesii* and *A*. *mariesii* in the NJ tree (S4 Fig). The three barcode combinations of *rbcL*+ *trnH-psbA* +*rps4-trnS* also failed to separate *A*. *gravesii* and *A*. *mariesii* in the NJ tree (S10 Fig), although the two species can be distinguished by a 19-bp insertion and a 2-bp inversion in *trnH-psbA* of *A*. *gravesii*. Considering the discriminatory power, cost-efficiency and effort, the two-barcode combination of *rbcL*+ *trnH-psbA* (Fig 4) seems to be the best choice for barcoding *Adiantum*. Furthermore, *trnL-F* had the highest variation and can be used as a barcoding marker at the intraspecific level.

### Need for nuclear barcodes for ferns

Chase *et al*. [2] suggested that multiple, low-copy nuclear markers with sufficient genetic variability and PCR-reliability need to be developed, which may permit researchers to detect hybrids. Four nuclear regions, the nuclear ribosomal internal transcribed spacer (ITS) region [62–63], the introns of the transcription factor *LEAFY* (*LFY*) [42,64–66], the cytosolic phosphoglucose isomerase gene *pgiC* [40,43,45,63,66–68], the two regions of the plastidicl glyceraldehydes-3-phosphate dehydrogenase (*gap*C*p*) -*gap*C*pSh* (*gap*C*p “short”*) [41,44,63,68–71], and *gap*C*pLg* (*gap*C*p “long”*) [71] were used to study the evolution of closely related fern species in recent years.

Rothfels *et al*. [70] presented 20 novel single-copy nuclear regions across ten distinct protein-coding genes: ApPEFP_C, cryptochrome 2 gene, cryptochrome 4 gene, *DET*1, *gap*C*p*S*h*, *IBR*3, *pgi*C, *SQD*1, *TPLATE*, and transducin gene which were readily amplified and sequenced from 15 diploid Polypodiales species. The results showed that *IBR*3_2, *DET*1, and *SQD*1_1 were amplified well, and clean sequences were obtained via direct Sanger sequencing from most taxa of Polypodiales examined.

Of the eight nuclear candidate markers (ITS, ITS2, *pgi*C, *gap*C, *LEAFY*, *IBR*3_2, *DET*1, and *SQD*1_1) screened in this study, ITS exhibited high PCR amplification success, but it did not produce a single band for PCR amplification due to additive banding and incomplete concerted evolution. ITS is thus difficult to be used as a barcode in ferns. The PCR amplification rates in six candidate nuclear regions (ITS2, *pgiC*, *gapCp*, *LEAFY*, *DET*1 and *SQD*1_1) were extremely low and/or clean sequences were not generated via direct sequencing without cloning.

Ishikawa *et al*. [40] developed primers to amplify the *pgiC* gene in ferns. The PCR products of primers 14F/16R containing two introns are moderate in size (534–1,000 bp) and the *pgiC* gene is possibly of value for phylogenetic reconstruction at the specific and generic levels. The primers 14F/15R and 15F/16R were developed and applied to study mating systems and other population genetic traits [40]. The *pgiC* gene was also used to detect the origins of polyploids and hybrids in *Dryopteris* [43,45,68]. But relatively universal primers of the *pgiC* gene are lacking [67].

Schneider *et al*. [63] employed three nuclear regions—ITS, *gapCp*, and *pgiC*, to explore patterns of reticulate evolution in *Asplenium*. The results showed that all three nuclear markers amplified well and several copies were recovered by cloning PCR products in *Asplenium*. Rothfels *et al*. [70] designed one novel primer pair for *pgiC* situated in exons 14 and 16, to amplify introns 14, 15, and exon 15 (about 600–700 bp); however, samples of *Adiantum* failed to be amplified and directly sequenced in their study.

We amplified *pgiC* using the primers of 14F/16R and 15F/16R, and obtained relatively weak bands in *Adiantum* when the 14F/16R primers were used. However, the sequences of *pgiC* are only about 300 bp in *Adiantum*, and showed multiple peaks in the sequencing signals. The primer pair 15F/16R failed to amplify taxa of *Adiantum*.

Schuettpelz *et al*. [41] designed primers to amplify part of the nuclear *gapCp* gene that encodes the glyceraldehyde-3-phosphate dehydrogenase. Their survey across ferns demonstrated that these primers are nearly universal for ferns, and holds considerable potential for addressing species-level questions across the tree of life in ferns. Rothfels *et al*. [70] designed specific primers for a region covering introns 8~10 of *gapCpSh*, which overlaps with the *gapCp* region amplified with the primers of Schuettpelz *et al*. [41], and ranges from 450 to 590 bp. In general *gapCpSh* amplified and sequenced well in fern taxa, however, they did not obtain clean sequences for *Adiantum* (only a partial 283 bp sequence of *A*. *pedatum* was obtained) and a few other genera (cloning not attempted). We amplified *gapCp* using the primers of Schuettpelz *et al*. [41], and obtained two or three bands in *Adiantum*.

*IBR*3_2 had a high PCR amplification rate even though it is difficult to obtain sequences via direct sequencing. It seems that clean sequences may be obtained from autoploid species whereas allopolyploid or hybrid ones failed. *IBR*3_2 successfully distinguished two presumed hybrid species—*A*. *meishanianum* from its maternal parent *A*. *malesianum* [72], and *A*. *ailaoshanense* [73] from its maternal parent *A*. *sinicum* by the degenerate base. The hybrid *A*. *meishanianum* [72] and its maternal *A*. *malesianum* had the same plastid sequences. Nevertheless, they can be distinguished in morphology and the *IBR*3_2 sequences. Therefore, *IBR*3_2 has the potential to be further explored as a nuclear barcode locus in some fern groups such as *Adiantum* and its close relatives.

### Taxonomic implications of the barcoding results in the context of morphology

DNA barcoding is generally successful for species identification in *Adiantum*, and the result is nearly congruent with morphology-based taxonomy except for a few species. Ultimately the systematics community relies on morphology for species delimitation [74–75]. Four species groups—*A*. *subpedatum* and *A*. *myriosorum*, *A*. *formosanum* and *A*. *refractum*, *A*. *juxtapositum* and *A*. *chienii*, *A*. *meishanianum* and *A*. *malesianum* cannot be identified by the plastid barcodes. We herein discuss these species groups in light of a morphological framework.

*Adiantum subpedatum* was recorded only from the Longtang Mountain in Zhejiang province [76], and was thought to be a possible depauperate form of *A*. *myriosorum* [30]. The primary differences between *A*. *subpedatum* and *A*. *myriosorum* lie in plant size and sori number [30]. The height of *A*. *subpedatum* is about 24–28 cm, whereas *A*. *myriosorum* is taller, about 40–60 cm. *Adiantum subpedatum* was described to have 1–2 sori per pinnule [76] while the latter has 4–6 sori. However, we noted that there are a few plants with 2–3 sori in some populations of *A*. *myriosorum*, while most samples from the two populations of the Longtang Mountain (type locality) of *A*. *subpedatum* have 3 sori per pinnule. Plant size and sori number may vary with the habitats. *Adiantum subpedatum* and *A*. *myriosorum* fell into the same clade in nuclear *IBR*3-2 tree (Fig 5). Based on the DNA barcoding results and our morphological observations, *A*. *subpedatum* is perhaps best treated as a synonym of *A*. *myriosorum*.

*Adiantum formosanum*, endemic to Taiwan, cannot be distinguished from *A*. *refractum*, because the two species have identical sequences of *trnL-F*, *rps*4*-trnS* (*rbcL* and *trnH-psbA* sequences are not available). Both species are epilithic plants, and have 2–4 sori on the thin papery and fan-shaped pinnules. The primary differences between *A*. *formosanum* and *A*. *refractum* are plant height and pinnule size. Although *A*. *formosanum* differ with *A*. *refractum* in a few nucleotides, it still fell into *A*. *refractum* clade in nuclear *IBR*3-2 tree (Fig 5). Based on the similar DNA sequences and the minor morphological differences, the species status of *A*. *formosanum* needs to be reevaluated and it may be treated as a synonym of *A*. *refractum*.

Lu *et al*. [77] proposed that *A*. *juxtapositum* might be a synonym of *A*. *chienii* based on chloroplast sequences and field observations. Samples of the Chinese *A*. *juxtapositum* and *A*. *chienii* clustered together in all NJ trees with a high BP value (Figs 2–4 and S2, S3 and S13 Figs). However, two samples of “*A*. *juxtapositum*” from Vietnam (CPC050 and CPC076 *A*. *sp*.) [78] did not cluster with the Chinese *A*. *juxtapositum*. The veins on lower surface of the samples from Vietnam are more visible than those of the Chinese samples. The fronds of the former are rather leathery while the latter are sub-leathery. The Chinese *A*. *juxtapositum* thus may be best treated as a synonym of *A*. *chienii* while the samples from Vietnam probably represent a new species.

*Adiantum meishanianum* was validated in 2009 [79]. A cryptic species related to *A*. *philippense* was suggested as its paternal species, and *A*. *malesianum* as its maternal parent [72]. The presumed hybrid species *A*. *meishanianum* and its maternal *A*. *malesianum* had identical plastid sequences. They can be distinguished by morphology (barely hirsute on rachis and lamina in *A*. *meishanianum* vs. densely hirsute on rachis and lamina in *A*. *malesianum*; pinnules with articulated stalks in *A*. *meishanianum* vs. pinnules nearly without stalks in *A*. *malesianum*). *Adiantum meishanianum* can also be distinguished from the maternal parent *A*. *malesianum* using *IBR*3_2.

Wang *et al*. [80] used the primers of Rothfels *et al*. [70] to amplify a region in the third exon of CRY2 and they detected a new hybrid species, *A*. *× ailaoshanense*, which probably originated from *A*. *sinicum* x *A*. *menglianense* [73]. However, the amplification and sequencing of CRY2 were not ideal in other series of *Adiantum* in our present study. Our *IBR*3_2 data also support the parents of the hybrid species *A*. *ailaoshanense* (*Yan 12413* and *Yan 12410*) to be *A*. *sinicum* and *A*. *menglianense* (Fig 5).

Mao *et al*. [37] placed *A*. *erythrochlamys* as a synonym of *A*. *roborowskii* var. *robustum*. The diagnostic characters between *A*. *roborowskii* and *A*. *erythrochlamys* are the number of false indusia (one vs. two) and the shape of pinnule margin (entire or undulate-crenate vs. bluntly serrate) [23]. The different states of the two characters can be observed in one population (WFH59) in Longnan, Gansu province, China. The samples with two false indusia and entire or undulate-crenate pinnule margins clustered with *A*. *roborowskii* var. *taiwanianum* whereas another sample was nested into another subclade in the *A*. *roborowskii* var. *roborowskii* clade. Our DNA barcoding results and morphology thus support the treatment of Mao *et al*. [37].

*Adiantum menglianense* was published in 1992 [38], and was included in the *Flora Yunnanica* treatment [81]. *Adiantum menglianense* formed a highly-supported clade, and was sister to the closely related species *A*. *philippense* with strong support (BP≥82) in all trees (Figs 2, 4 and 5 and S2–S13 Figs) except for the *trnH-psbA* tree (the closely related species of *A*. *menglianense* is *A*. *capillus-junonis* in Fig 3). The margin of pinnules is deeply lobed in *A*. *menglianense* (vs. subentire in *A*. *philippense*); the false indusia are short and straight in *A*. *menglianense* (vs. long and cupped in *A*. *philippense*); and pinnules are palmate and thin in *A*. *menglianense* (vs. semilunar and thick in *A*. *philippense*). *Adiantum menglianense* was described to have 6–10 sori per pinnule [38] while *A*. *philippense* has 2–6 sori [30]. Zhang *et al*. [72] pointed out that a cryptic species under *A*. *philippense* is the paternal parent of *A*. *meishanianum*. The present analyses confirmed the paternal species of *A*. *meishanianum* as *A*. *menglianense*.

*Adiantum gravesii* and *A*. *mariesii* failed to be identified except by *trnH-psbA* and *IBR*3_2. *Adiantum gravesii* can be distinguished from *A*. *mariesii* using morphological characters with the former being a bigger plant with reniform or lunate indusia vs. the latter a smaller plant with circular indusia.

## Conclusions and Outlook

Due to low primer universality of *matK* and the existence of multiple copies of ITS, these two commonly used barcodes were not appropriate for *Adiantum*. With the consideration of discriminatory power, cost-efficiency and effort, the two-barcode combination of *rbcL*+ *trnH-psbA* seems to be the best choice for barcoding *Adiantum*, and perhaps basal polypod ferns in general. Coupling DNA barcoding with morphology provides important insights into species delimitations for several taxa in our case study. Overall DNA barcoding provides additional DNA diagnostic characters to discriminate the individuals lacking diagnostic features because of rapid diversification, morphological stasis, and phenotypic variations [82]. Because hybridizations and allopolyploidy are common in ferns, we argue for including a selected group of nuclear loci as barcodes, especially via the next-generation sequencing, as it is more efficient and economical to obtain single-copy nuclear loci without the cloning procedure [9, 83–84]. With the drastic decrease in cost with the next-generation sequencing, fern barcoding can also effectively incorporate whole plastome data as organelle barcodes (e.g., [85]).

## Supporting Information

S1 Fig Agarose gel electrophoresis of PCR products of *IBR*3_2. (PDF)

S2 Fig The NJ tree based on *rps4-trnS* marker using p-distance model. (PDF)

S3 Fig The NJ tree based on *trnL-F* sequences using the *p*-distance model. (PDF)

S4 Fig The NJ tree based on the combination of *rps4-trnS + rbcL*. (PDF)

S5 Fig The NJ tree based on the combination of *trnL-F + rps4-trnS*. (PDF)

S6 Fig The NJ tree based on the combination of *trnL-F + rbcL*. (PDF)

S7 Fig The NJ tree based on the combination of *trnH-psbA + rps4-trnS*. (PDF)

S8 Fig The NJ tree based on the combination of *trnL-F + trnH-psbA*. (PDF)

S9 Fig The NJ tree based on the combination of *trnL-F + trnH-psbA + rps4-trnS*. (PDF)

S10 Fig The NJ tree based on the combination of *trnH-psbA + rps4-trnS + rbcL*. (PDF)

S11 Fig The NJ tree based on the combination of *trnL-F + rps4-trnS* + *rbcL*. (PDF)

S12 Fig The NJ tree based on the combination of *trnL-F + trnH-psbA + rbcL*. (PDF)

S13 Fig The NJ tree based on the combination of *trnL-F + trnH-psbA + rps4-trnS + rbcL*. (PDF)

S1 Table Taxa, voucher specimens and GenBank Accession Numbers in this study. (DOCX)

S2 Table Taxa, voucher specimens and GenBank Accession Numbers of *IBR*3_2 sequences. (DOCX)

S3 Table Primers used in this study. (DOCX)
