# Supplementary figures and images for: Applying DNA Barcodes to Identify Closely Related Species of Ferns: A Case Study of the Chinese Adiantum (Pteridaceae)
Source: PLoS One. 2016 Sep 7;11(9):e0160611. doi: 10.1371/journal.pone.0160611 (PMC5014338; doi:10.1371/journal.pone.0160611)

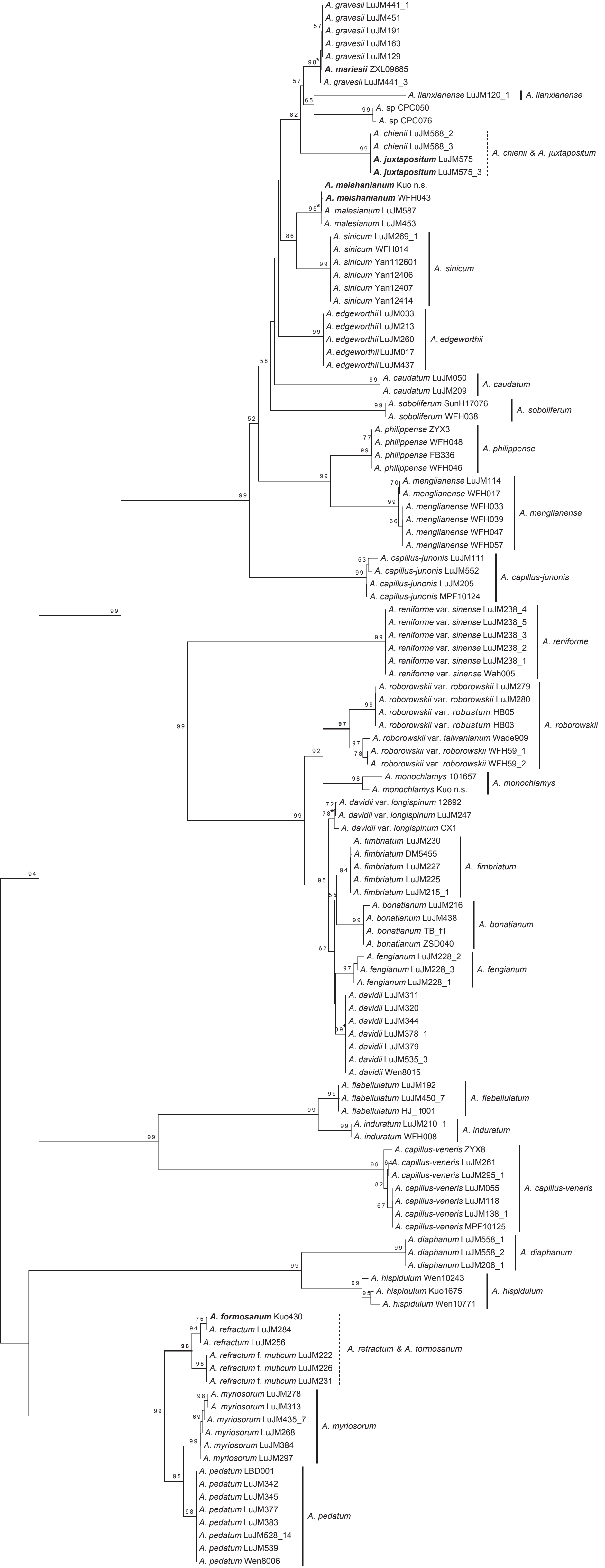

Supplement: S2 Fig — (PDF) [file pone.0160611.s002.pdf]

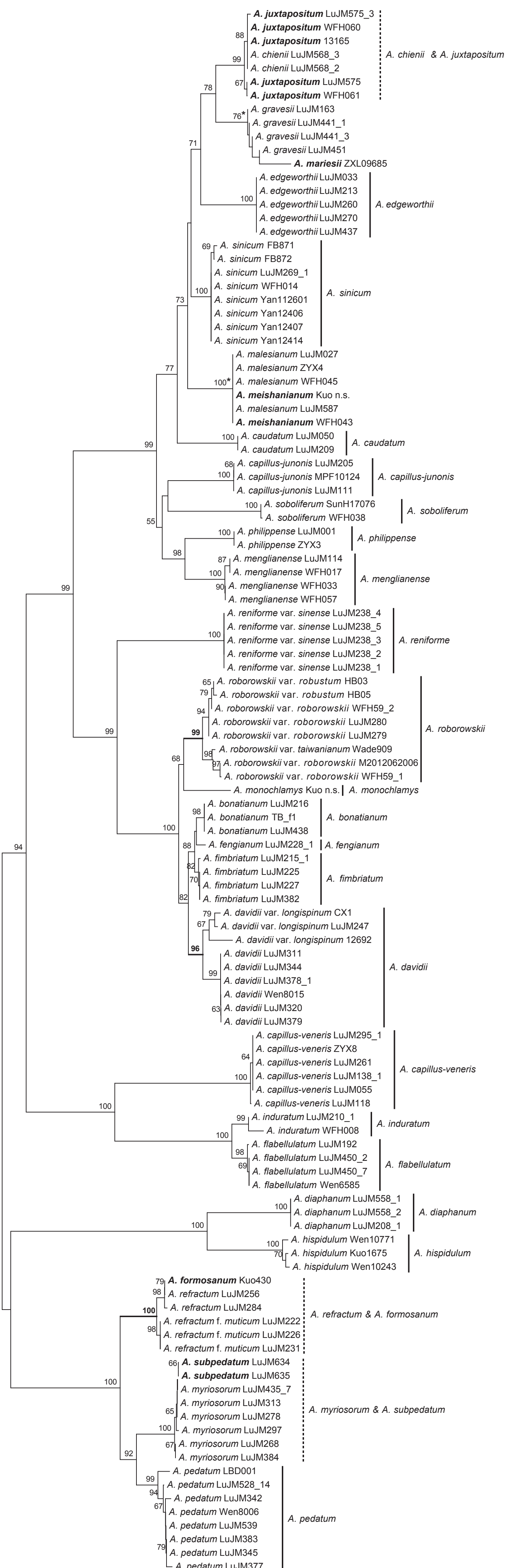

Supplement: S3 Fig — (PDF) [file pone.0160611.s003.pdf]

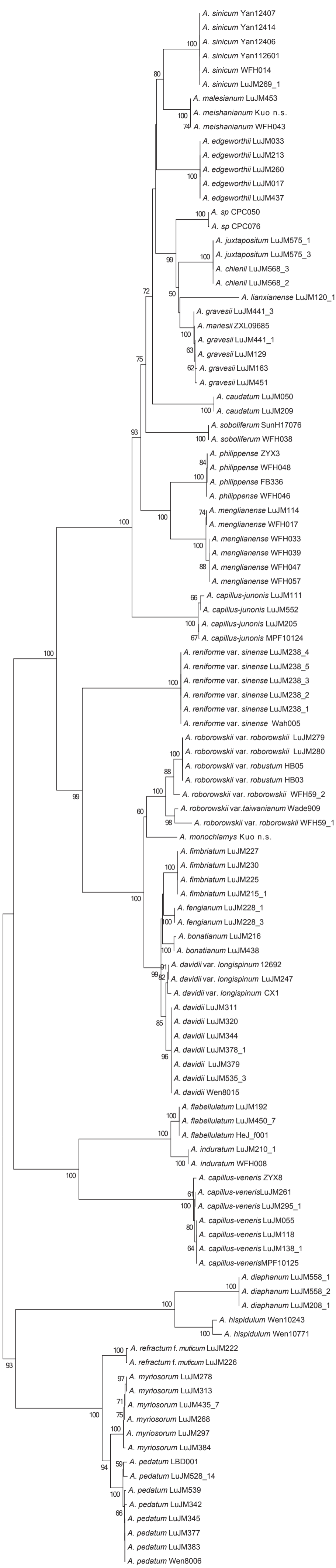

0.01

Supplement: S4 Fig — (PDF) [file pone.0160611.s004.pdf]

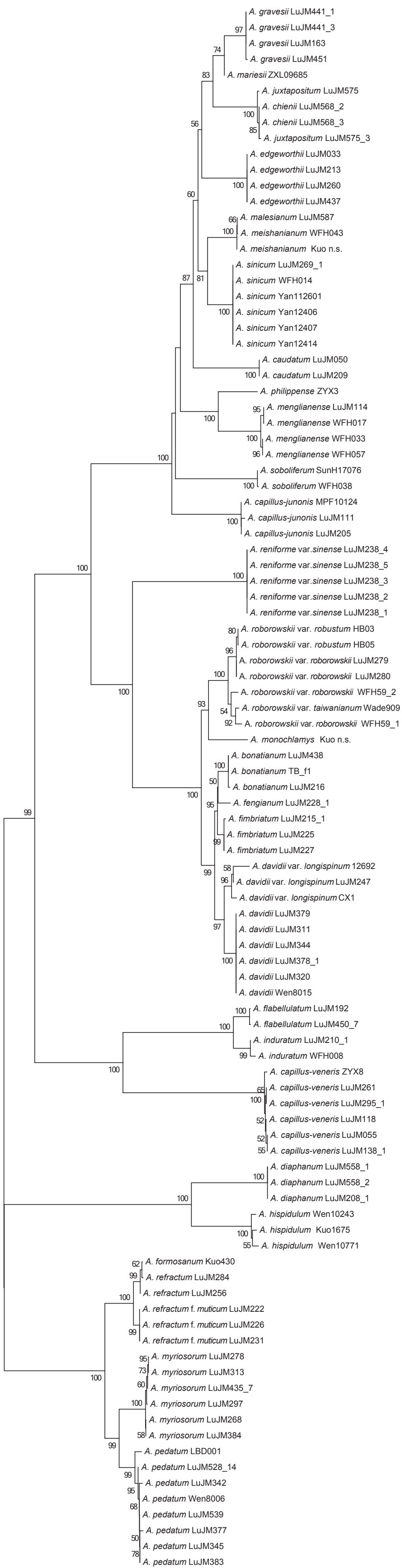

0.02

Supplement: S5 Fig — (PDF) [file pone.0160611.s005.pdf]

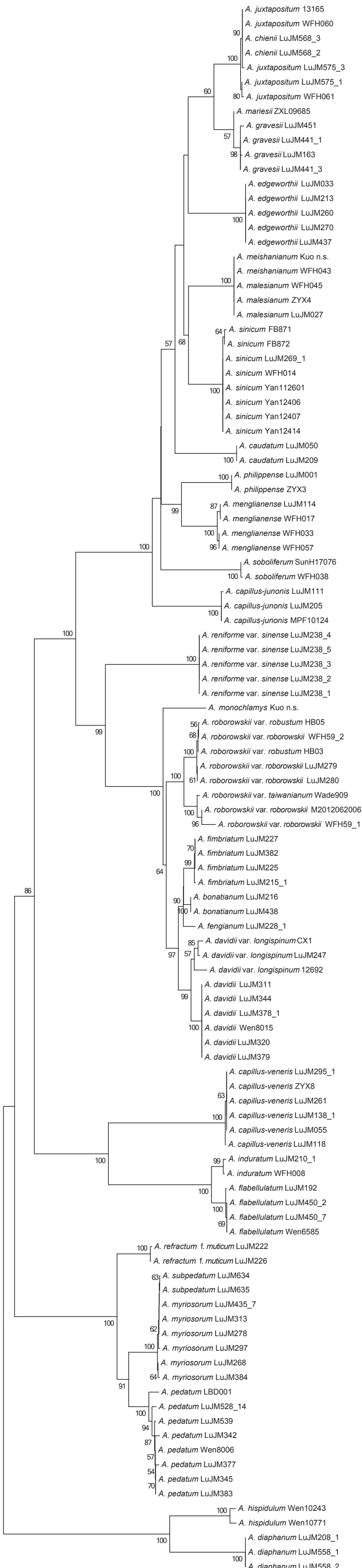

0.01

Supplement: S6 Fig — (PDF) [file pone.0160611.s006.pdf]

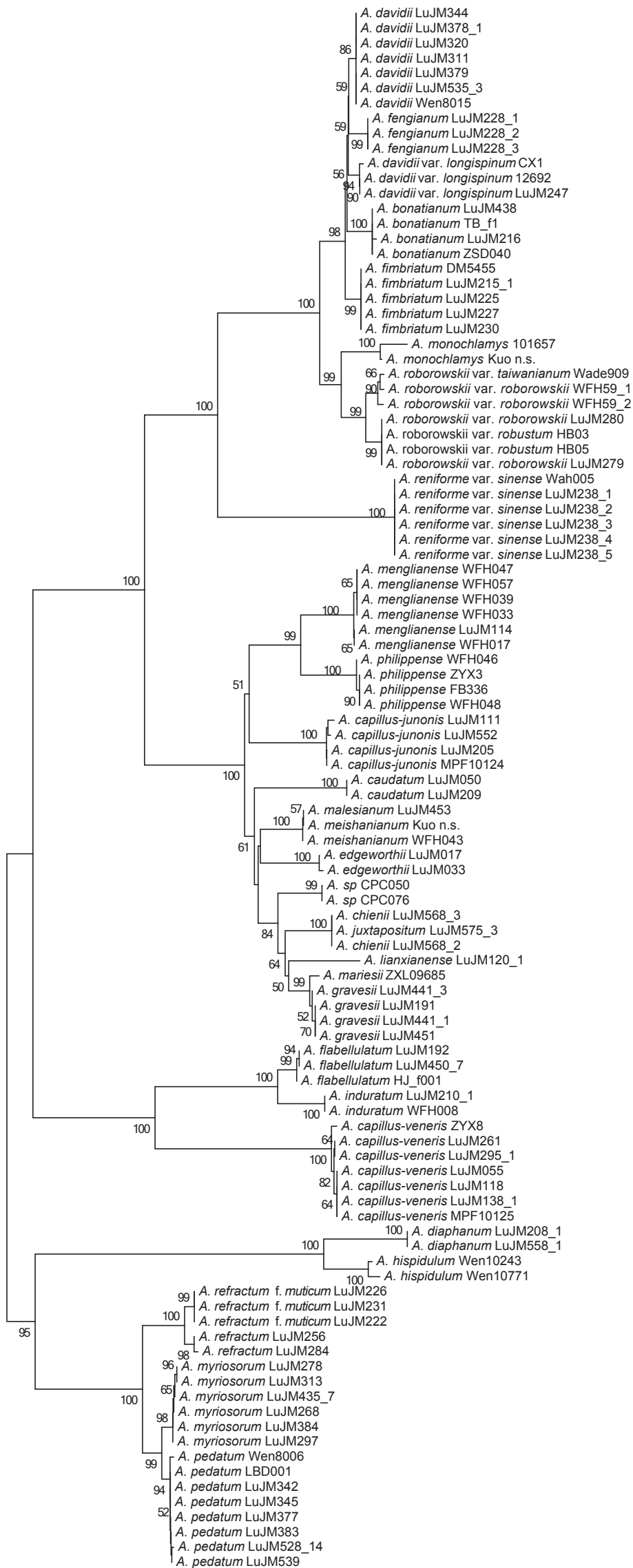

0.01

Supplement: S7 Fig — (PDF) [file pone.0160611.s007.pdf]

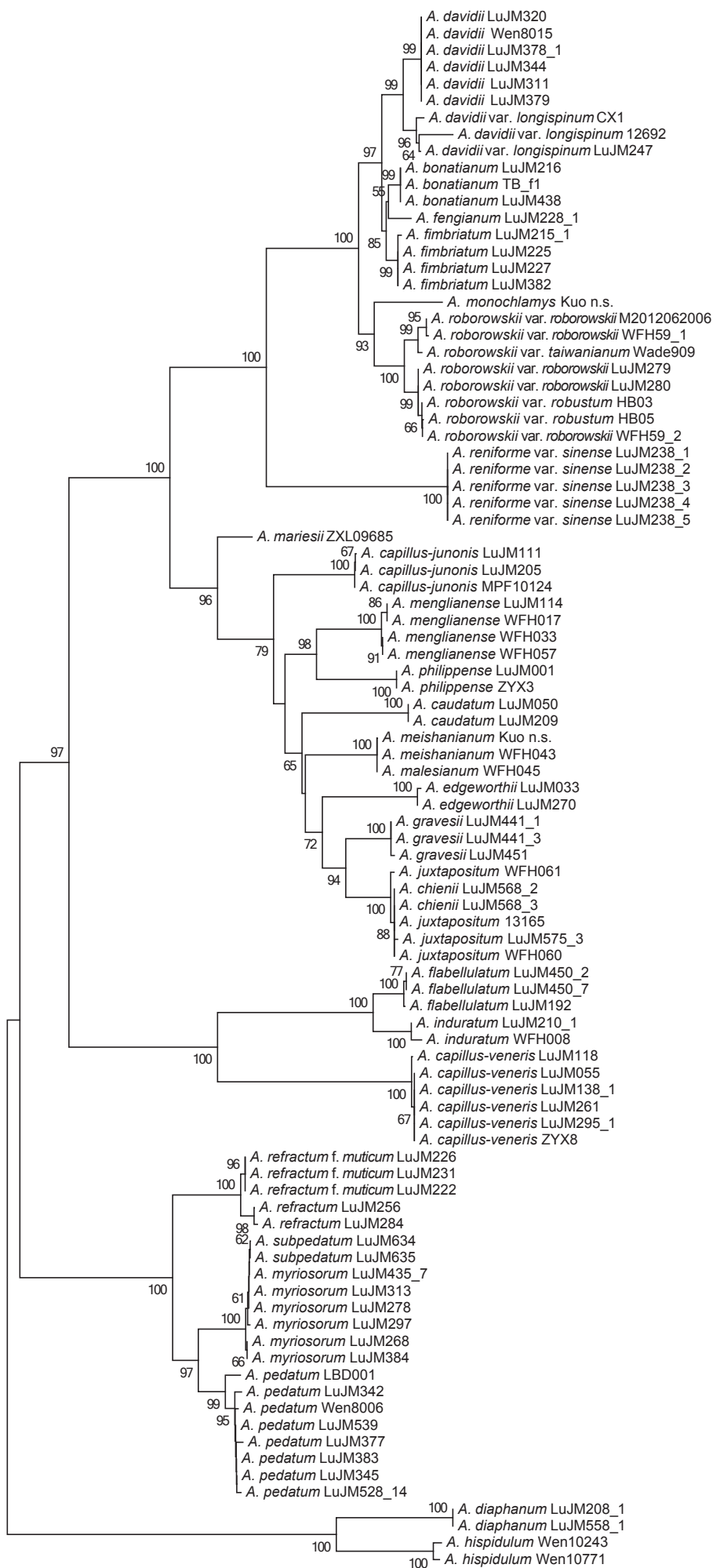

0.02

Supplement: S8 Fig — (PDF) [file pone.0160611.s008.pdf]

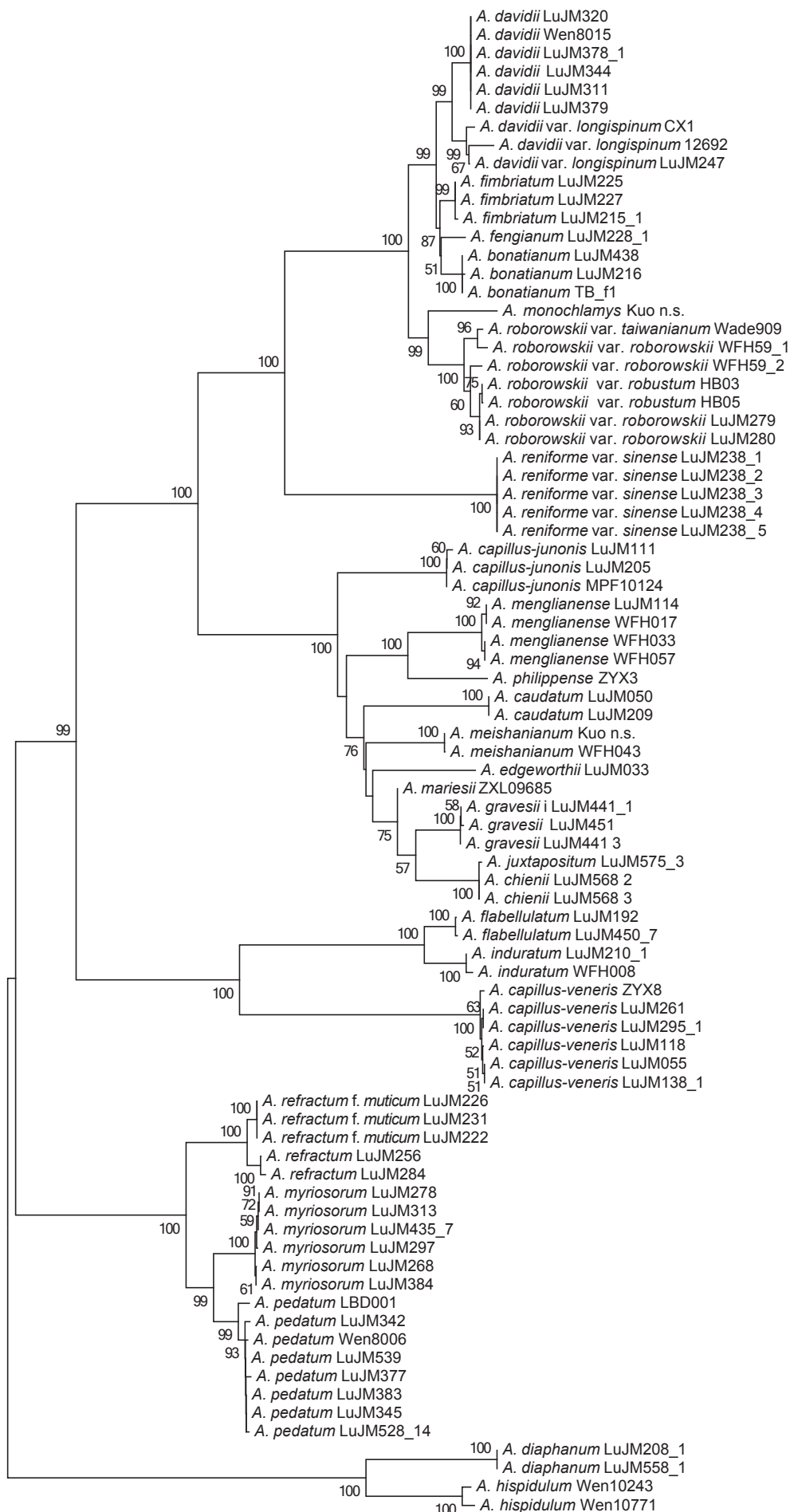

0.02

Supplement: S9 Fig — (PDF) [file pone.0160611.s009.pdf]

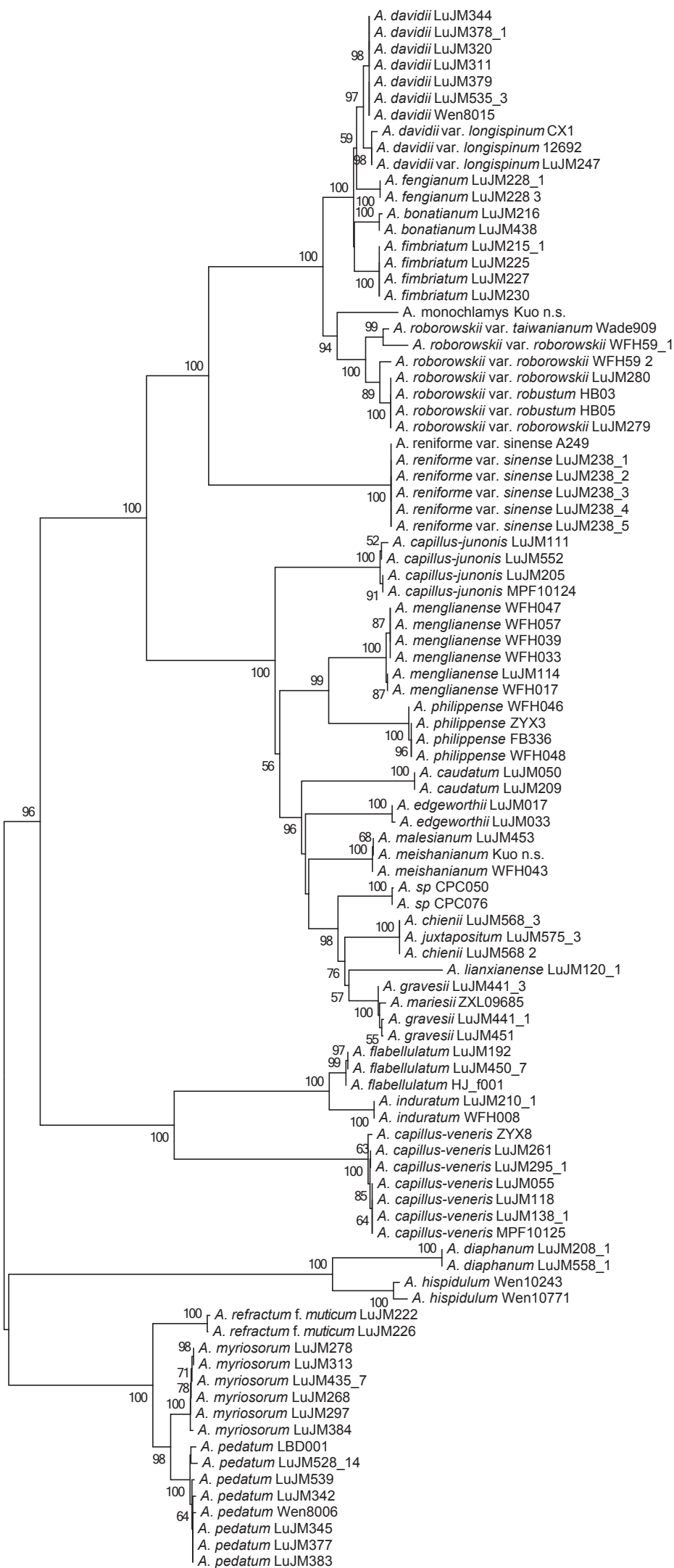

0.01

Supplement: S10 Fig — (PDF) [file pone.0160611.s010.pdf]

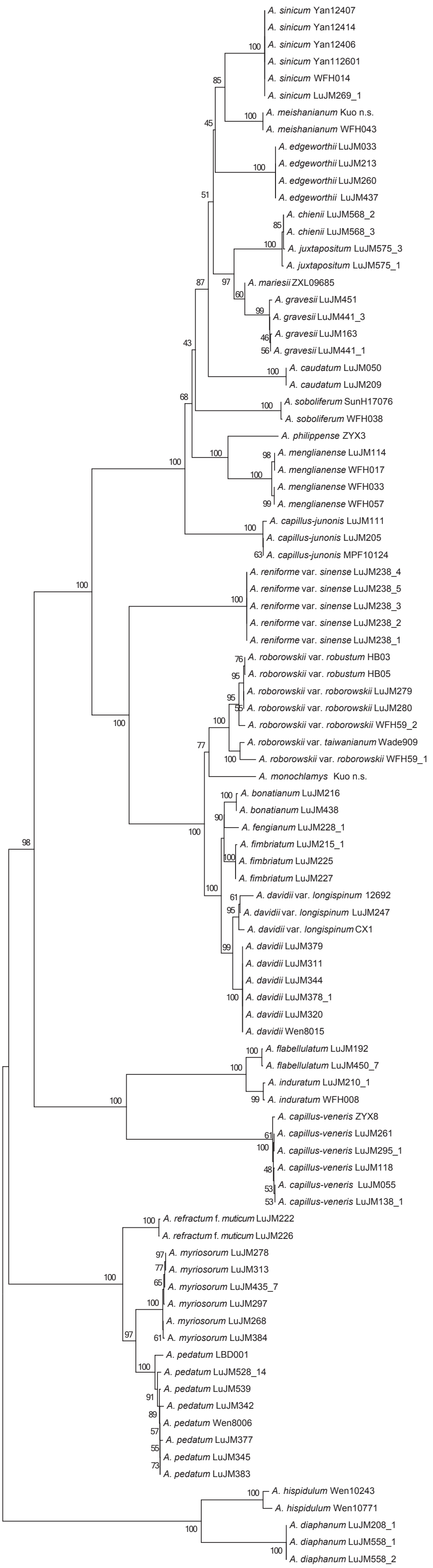

0.02

Supplement: S11 Fig — (PDF) [file pone.0160611.s011.pdf]

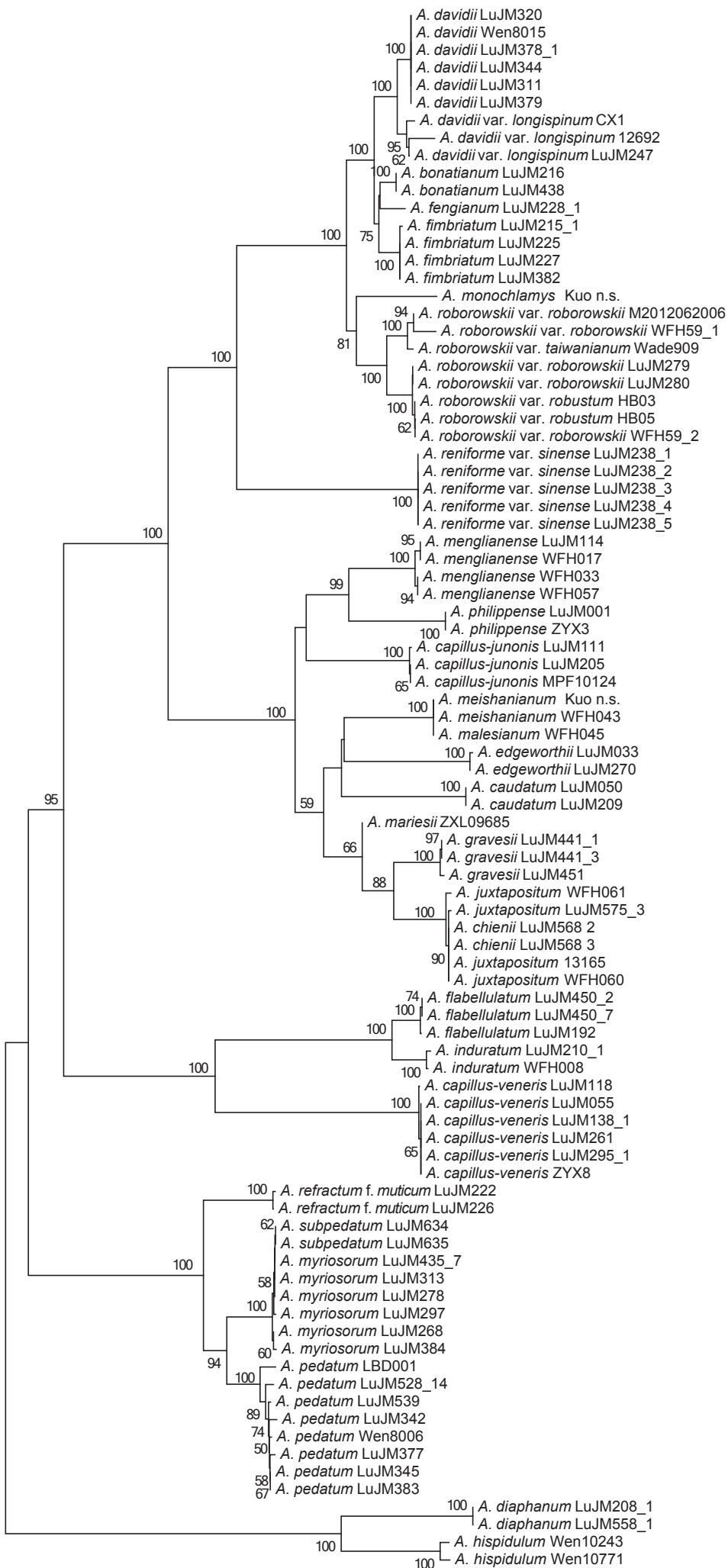

0.01

Supplement: S12 Fig — (PDF) [file pone.0160611.s012.pdf]

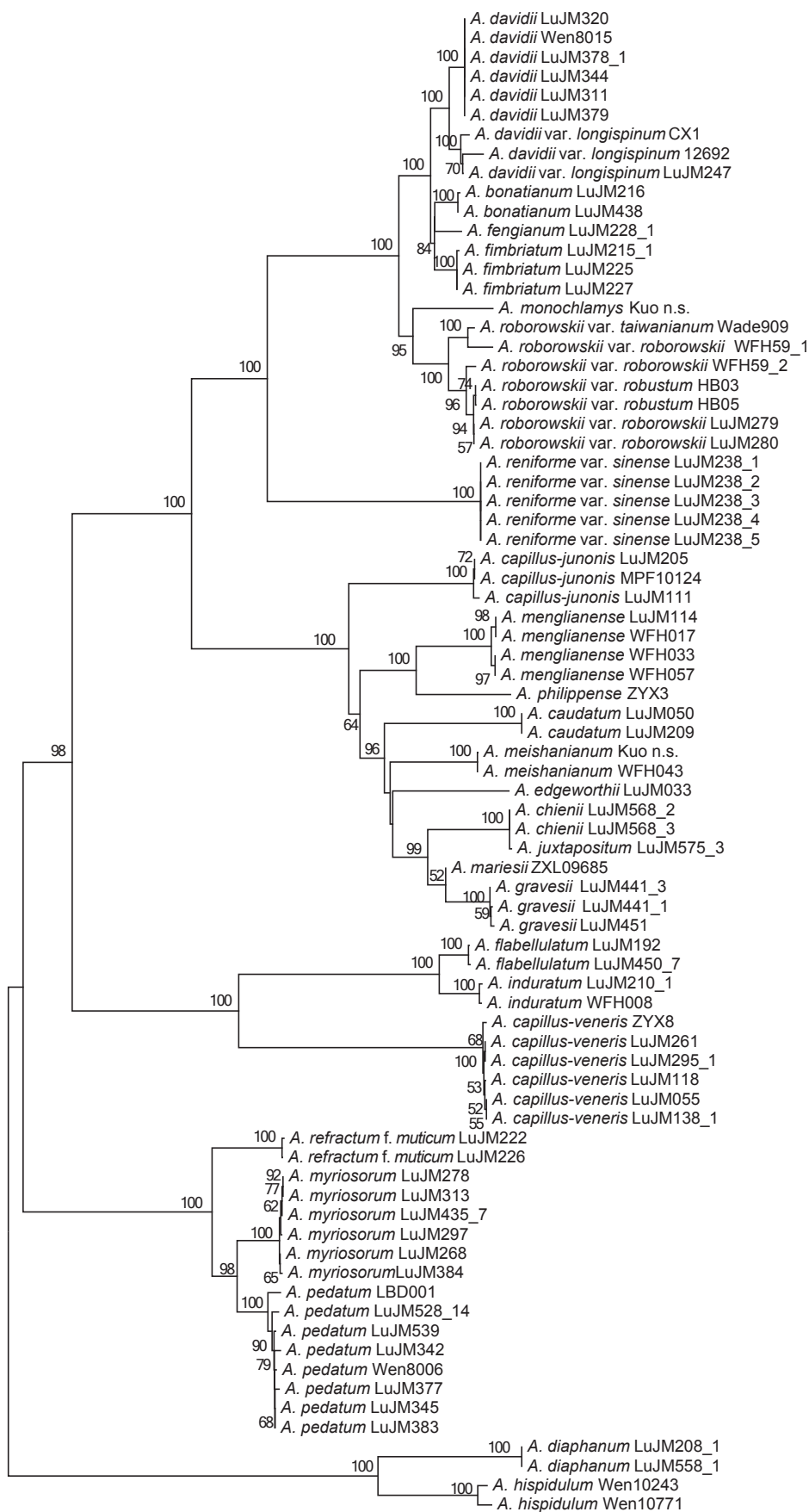

0.01

Supplement: S13 Fig — (PDF) [file pone.0160611.s013.pdf]
