# Supplementary material for: Applying DNA Barcodes to Identify Closely Related Species of Ferns: A Case Study of the Chinese Adiantum (Pteridaceae)
Source: PLoS One. 2016 Sep 7;11(9):e0160611. doi: 10.1371/journal.pone.0160611 (PMC5014338; doi:10.1371/journal.pone.0160611)
Supplement: S1 Table — (DOCX) [file pone.0160611.s014.docx]

Table S1 Taxa, voucher specimens and GenBank accession numbers in this study.

| Taxon | Herbarium/  Voucher number | **GenBank Accession Numbers** | | | |
| --- | --- | --- | --- | --- | --- |
|  |  | ***rbc*L** | ***trn*H*-psb*A** | ***trn*L-F** | ***rps4-trn*S** |
| *A. bonatianum* Brause | KUN FB882 | KT426912 | KT427102 | - | - |
| *A. bonatianum* Brause | KUN LuJM216 | JF935294 | KT427103 | JF980639 | JF980556 |
| *A. bonatianum* Brause | KUN LuJM438 | KT426913 | KT427104 | KT427023 | KT427258 |
| *A. bonatianum* Brause | KUN TB_f1 | - | KT427105 | KT427024 | KT427259 |
| *A. bonatianum* Brause | KUN ZSD40 | - | KT427106 | - | KT427260 |
| *A. capillus-junonis* Rupr. | KUN LuJM111 | JF935314 | KT427107 | JF980662 | JF980578 |
| *A. capillus-junonis* Rupr. | KUN LuJM205 | KT426914 | KT427108 | KT427025 | KT427261 |
| *A. capillus-junonis* Rupr. | KUN LuJM552 | KT426915 | KT427109 | - | KT427262 |
| *A. capillus-junonis* Rupr. | KUN MPF10124 | KT426916 | KT427110 | KT427026 | KT427263 |
| *A. capillus-veneris* L. | KUN LuJM055 | KT426917 | KT427111 | KT427027 | KT427264 |
| *A. capillus-veneris* L. | KUN LuJM118 | JF935318 | KT427112 | JF980665 | JF980582 |
| *A. capillus-veneris* L. | KUN LuJM138_1 | JF935320 | KT427113 | JF980666 | JF980584 |
| *A. capillus-veneris* L. | KUN LuJM261 | KT426918 | KT427115 | KT427029 | KT427267 |
| *A. capillus-veneris* L. | KUN LuJM295_1 | JF935322 | KT427116 | JF980667 | JF980586 |
| *A. capillus-veneris* L. | KUN ZYX8 | KT426924 | KT427124 | KT427030 | KT427272 |
| *A. capillus-veneris* L. | KUN MPF10125 | KT426925 | KT427125 | - | KT427273 |
| *A. caudatum* L. | KUN LuJM050 | JF935297 | KT427127 | JF980642 | JF980559 |
| *A. caudatum* L. | KUN LuJM209 | JF935296 | KT427128 | JF980641 | JF980558 |
| *A. caudatum* L. | KUN LuJM211 | KT426927 | KT427129 | - | - |
| *A. caudatum* L. | KUN ZYX6 | KT426928 | KT427130 | - | - |
| *A. caudatum* L. | KUN WFH056 | KT426929 | KT427132 | - | - |
| *A. chienii* Ching | KUN LuJM568_2 | KT426959 | KT427179 | KT427059 | KT427302 |
| *A. chienii* Ching | KUN LuJM568_3 | JF935303 | KT427180 | JF980649 | JF980565 |
| *A. davidii* var. *davidii* Franch. | KUN LuJM311 | KT426930 | KT427133 | KT427031 | KT427274 |
| *A. davidii* var. *davidii* Franch. | KUN LuJM320 | KT426931 | KT427134 | KT427032 | KT427275 |
| *A. davidii* var. *davidii* Franch. | KUN LuJM344 | JF935310 | KT427135 | JF980659 | JF980574 |
| *A. davidii* var. *davidii* Franch. | KUN LuJM378 | KT426932 | KT427136 | KT427033 | KT427276 |
| *A. davidii* var. *davidii* Franch. | KUN LuJM379 | KT426933 | KT427137 | KT427034 | KT427277 |
| *A. davidii* var. *davidii* Franch. | KUN LuJM535 | KT426935 | KT427139 | - | KT427278 |
| *A. davidii* var. *davidii* Franch. | US Wen8015 | JF935319 | KT427143 | KT427038 | JF980583 |
| *A. davidii* var. *longispinum* Ching | CSH 12692 | KT426936 | KT427140 | KT427036 | KT427279 |
| *A. davidii* var. *longispinum* Ching | KUN CX1 | JF935316 | KT427141 | KT427037 | JF980580 |
| *A. davidii* var. *longispinum* Ching | KUN LuJM247 | JF935292 | KT427142 | JF980638 | JF980554 |
| *A. diaphanum* Blume | KUN LuJM208_1 | JF935301 | KT427144 | JF980647 | JF980563 |
| *A. diaphanum* Blume | KUN LuJM558_1 | JF935304 | KT427146 | JF980650 | JF980566 |
| *A. diaphanum* Blume | KUN LuJM558_2 | KT426937 | - | KT427039 | KT427280 |
| *A. edgeworthii* Hook. | KUN LuJM017 | KT426938 | KT427147 | - | KT427283 |
| *A. edgeworthii* Hook. | KUN LuJM033 | KT426940 | KT427148 | KT427041 | KT427284 |
| *A. edgeworthii* Hook. | KUN LuJM213 | KT426941 | - | KT427042 | KT427285 |
| *A. edgeworthii* Hook. | KUN LuJM260 | KT426942 | - | KT427043 | KT427286 |
| *A. edgeworthii* Hook. | KUN LuJM270 | KT426943 | KT427149 | KT427044 | - |
| *A. edgeworthii* Hook. | KUN LuJM437 | KT426944 | - | KT427045 | KT427287 |
| *A. fengianum* Ching | KUN LuJM228_1 | JF935308 | KT427150 | JF980656 | JF980571 |
| *A. fengianum* Ching | KUN LuJM228_2 | - | KT427151 | - | KT427288 |
| *A. fengianum* Ching | KUN LuJM228_3 | KT426945 | KT427152 | - | KT427289 |
| *A. fimbriatum* Christ | CSH DM5455 | - | KT427250 | - | KT427354 |
| *A. fimbriatum* Christ | KUN LuJM215_1 | JF935321 | KT427154 | KT427046 | JF980585 |
| *A. fimbriatum* Christ | KUN LuJM225 | KT426948 | KT427156 | KT427047 | KT427291 |
| *A. fimbriatum* Christ | KUN LuJM227 | JF935293 | KT427157 | KT427048 | JF980555 |
| *A. fimbriatum* Christ | KUN LuJM230 | KT426949 | KT427158 | - | KT427292 |
| *A. fimbriatum* Christ | KUN LuJM382 | KT426951 | KT427160 | KT427051 | - |
| *A. flabellulatum* L. | KUN HeJ_f001 | KT426952 | KT427161 | - | KT427295 |
| *A. flabellulatum* L. | KUN LuJM192 | JF935315 | KT427163 | JF980663 | JF980579 |
| *A. flabellulatum* L. | KUN LuJM450_2 | KT426954 | KT427164 | KT427052 | - |
| *A. flabellulatum* L. | KUN LuJM450_7 | JF935295 | KT427165 | JF980640 | JF980557 |
| *A. flabellulatum* L. | US Wen6585 | JF935325 | - | JF980670 | JF980589 |
| *A. formosanum* Tagawa | Kuo 430 | - | - | JF980652 | JF980568 |
| *A. gravesii* Hance | KUN LuJM129 | JF935312 | - | - | JF980576 |
| *A. gravesii* Hance | KUN LuJM163 | JF935313 | - | JF980661 | JF980577 |
| *A. gravesii* Hance | KUN LuJM191 | - | KT427166 | - | KT427298 |
| *A. gravesii* Hance | KUN LuJM441_1 | JF935306 | KT427167 | JF980653 | JF980569 |
| *A. gravesii* Hance | KUN LuJM441_3 | KT426955 | KT427168 | KT427056 | KT427299 |
| *A. gravesii* Hance | KUN LuJM451 | JF935317 | KT427170 | JF980664 | JF980581 |
| *A. hispidulum* Sw. | TAIF Kuo1675 | - | - | JF980645 | KT427300 |
| *A. hispidulum* Sw. | US Wen10243 | JF935341 | KT427171 | JF980686 | JF980607 |
| *A. hispidulum* Sw. | US Wen10771 | JF935349 | KT427172 | JF980694 | JF980615 |
| *A. induratum* Christ | KUN LuJM210_1 | JF935309 | KT427173 | JF980657 | JF980572 |
| *A. induratum* Christ | KUN WFH008 | KT426956 | KT427177 | KT427057 | KT427301 |
| *A. juxtapositum*Ching | CSH 13165 | KT426957 | KT427178 | KT427058 | - |
| *A. juxtapositum* Ching | KUN LuJM575_1 | KT426960 | - | KT427060 | KT427303 |
| *A. juxtapositum* Ching | KUN LuJM575_3 | JF935305 | KT427181 | JF980651 | JF980567 |
| *A. juxtapositum* Ching | KUN WFH060 | KT426961 | KT427182 | KT427061 | - |
| *A. juxtapositum* Ching | KUN WFH061 | KT426962 | KT427183 | KT427062 | - |
| *A. lianxianense* Ching & Y. X. Lin in Y. X. Lin | KUN LuJM120 | JF935302 | KT427184 | - | JF980564 |
| *A. malesianum* J. Ghatak | KUN LuJM027 | KT426963 | - | KT427063 | - |
| *A. malesianum* J. Ghatak | KUN LuJM453 | KT426964 | KT427185 | - | KT427304 |
| *A. malesianum* J. Ghatak | KUN LuJM587 | - | - | KT427065 | KT427305 |
| *A. malesianum* J. Ghatak | KUN ZYX4 | KT426966 | - | KT427066 | - |
| *A. malesianum* J. Ghatak | KUN WFH045 | KT426968 | KT427187 | KT427067 | - |
| *A. mariesii* Baker | CSH ZXL09685 | KT426970 | KT427188 | - | KT427307 |
| *A. meishanianum* F.S. Hsu ex Y.C. Liu & W.L. Chiou | KUN WFH043 | KT426972 | KT427190 | KT427069 | KT427309 |
| *A. meishanianum* F.S. Hsu ex Y.C. Liu & W.L. Chiou | TAIF Kuo n.s. | KT426971 | KT427189 | KT427068 | KT427308 |
| *A. menglianense* Y.Y. Qian | KUN LuJM114 | JF935311 | KT427191 | JF980660 | JF980575 |
| *A. menglianense* Y.Y. Qian | KUN WFH017 | KT426973 | KT427192 | KT427070 | KT427310 |
| *A. menglianense* Y.Y. Qian | KUN WFH033 | KT426974 | KT427193 | KT427071 | KT427311 |
| *A. menglianense* Y.Y. Qian | KUN WFH039 | KT426975 | KT427194 | - | KT427312 |
| *A. menglianense* Y.Y. Qian | KUN WFH047 | KT426976 | KT427195 | - | KT427313 |
| *A. menglianense* Y.Y. Qian | KUN WFH057 | KT426977 | KT427196 | KT427072 | KT427314 |
| *A. monochlamys* D.C. Eaton | TAIF Kuo n.s. | KT426978 | KT427198 | KT427073 | KT427316 |
| *A. monochlamys* D.C. Eaton | TNS 764001 | - | KT427197 | - | KT427315 |
| *A. monochlamys* D.C. Eaton^#^ | TNS 764001 | AB574800 | AB575453 | - | - |
| *A. myriosorum* Baker | KUN LuJM268 | KT426979 | KT427199 | KX387409 | KT427317 |
| *A. myriosorum* Baker | KUN LuJM278 | KT426980 | KT427200 | KX387406 | KT427318 |
| *A. myriosorum* Baker | KUN LuJM297 | JF935359 | KT427201 | JF980706 | JF980628 |
| *A. myriosorum* Baker | KUN LuJM313 | KT426981 | KT427202 | KX387408 | KX387421 |
| *A. myriosorum* Baker | KUN LuJM384 | KT426982 | KT427203 | KX387410 | KX387420 |
| *A. myriosorum* Baker | KUN LuJM435 | KT426983 | KT427204 | KX387407 | KT427319 |
| *A. pedatum* L. | KUN LBD001 | KT426987 | KT427209 | KT427078 | KT427321 |
| *A. pedatum* L. | KUN LuJM342 | KT426988 | KT427210 | KT427079 | KT427322 |
| *A. pedatum* L. | KUN LuJM343 | JF935360 | KT427211 | JF980707 | JF980629 |
| *A. pedatum* L. | KUN LuJM345 | KT426989 | KT427212 | KX387411 | KX387416 |
| *A. pedatum* L. | KUN LuJM377 | KT426990 | KT427213 | KX387412 | KX387418 |
| *A. pedatum* L. | KUN LuJM383 | KT426991 | KT427214 | KX387413 | KX387419 |
| *A. pedatum* L. | KUN LuJM528 | KT426992 | KT427216 | KT427080 | KT427324 |
| *A. pedatum* L. | KUN LuJM539 | KT426994 | KT427218 | KT427081 | KT427325 |
| *A. pedatum* L. | KUN Wen8006 | KT426995 | KT427219 | KT427082 | KX387417 |
| *A. philippense* L. | KUN FB336 | KT426996 | KT427220 | - | KT427326 |
| *A. philippense* L. | KUN LuJM001 | JF935331 | KT427221 | KT427083 | JF980596 |
| *A. philippense* L. | KUN ZYX3 | KT426997 | KT427223 | KT427084 | KT427329 |
| *A. philippense* L. | KUN ZYX9 | JF935299 | KT427225 | JF980644 | JF980561 |
| *A. philippense* L. | KUN WFH046 | KT427000 | KT427228 | - | KT427334 |
| *A. philippense* L. | KUN WFH048 | KT427001 | KT427229 | - | KT427335 |
| *A. refractum* f. *muticum* (Ching) Y. X. Lin | KUN LuJM222 | JF935291 | KT427232 | JF980637 | JF980553 |
| *A. refractum* f. *muticum* (Ching) Y. X. Lin | KUN LuJM226 | JF935290 | KT427233 | JF980636 | JF980552 |
| *A. refractum* f. *muticum* (Ching) Y. X. Lin | KUN LuJM231 | - | KT427234 | KT427085 | KT427336 |
| *A. refractum* Christ | KUN LuJM256 | - | KT427236 | KT427086 | KT427337 |
| *A. refractum* Christ | KUN LuJM284 | - | KT427237 | KT427087 | KT427338 |
| *A. reniforme* L. var. *sinense* Y.X. Lin | IBSC Wah005 | KT427004 | KT427241 | - | KT427342 |
| *A. reniforme* L. var. *sinense* Y.X. Lin | KUN LuJM238_1 | JF935287 | KT427243 | JF980633 | JF980549 |
| *A. reniforme* L. var. *sinense* Y.X. Lin | KUN LuJM238_2 | KT427005 | KT427244 | KT427088 | KT427344 |
| *A. reniforme* L. var. *sinense* Y.X. Lin | KUN LuJM238_3 | KT427006 | KT427245 | KT427089 | KT427345 |
| *A. reniforme* L. var. *sinense* Y.X. Lin | KUN LuJM238_4 | KT427007 | KT427246 | KT427090 | KT427346 |
| *A. reniforme* L. var. *sinense* Y.X. Lin | KUN LuJM238_5 | KT427008 | KT427247 | KT427091 | KT427347 |
| *A. roborowskii* Maxim var. *robustum* Christ | BJFC HB03 | KT427018 | KT427251 | KT427097 | KT427355 |
| *A. roborowskii* Maxim var. *robustum* Christ | BJFC HB05 | KT427019 | KT427252 | KT427098 | KT427356 |
| *A. roborowskii* var. *roborowskii* Maxim. | BJFC M2012062006 | KT427020 | KT427255 | KT427099 | - |
| *A. roborowskii* var. *roborowskii* Maxim. | KUN LuJM279 | JF935289 | KT427253 | JF980635 | JF980551 |
| *A. roborowskii* var. *roborowskii* Maxim. | KUN LuJM280 | JF935298 | KT427254 | JF980643 | JF980560 |
| *A. roborowskii* var. *roborowskii* Maxim. | KUN WFH59_1 | KT427021 | KT427256 | KT427100 | KT427357 |
| *A. roborowskii* var. *roborowskii* Maxim. | KUN WFH59_2 | KT427022 | KT427257 | KT427101 | KT427358 |
| *A. roborowskii* Maxim. var. *taiwanianum* (Tagawa) W.C. Shieh | TAIF Wade909 | KT427009 | KT427248 | JF980654 | KT427348 |
| *A. sinicum* Ching^#^ | CSH Yan112601 | KP637240 | - | KP637270 | KP637255 |
| *A. sinicum* Ching^#^ | CSH Yan12406 | KP637237 | - | KP637267 | KP637252 |
| *A. sinicum* Ching^#^ | CSH Yan12407 | KP637238 | - | KP637268 | KP637253 |
| *A. sinicum* Ching^#^ | CSH Yan12414 | KP637239 | - | KP637269 | KP637254 |
| *A. sinicum* Ching | KUN FB871 | KT427011 | - | KT427092 | - |
| *A. sinicum* Ching | KUN FB872 | KT427012 | - | KT427093 | - |
| *A. sinicum* Ching | KUN LuJM269_1 | JF935300 | - | JF980646 | JF980562 |
| *A. sinicum* Ching | KUN WFH014 | KT427015 | - | KT427094 | KT427351 |
| *A. soboliferum* Wall. ex Hook. | KUN SunH17076 | KT427016 | - | KT427095 | KT427352 |
| *A. soboliferum* Wall. ex Hook. | KUN WFH038 | KT427017 | - | KT427096 | KT427353 |
| *A. sp.* | KUN CPC050 | KX387401 | KX387403 | - | KX387414 |
| *A. sp.* | KUN CPC076 | KX387402 | KX387404 | - | KX387415 |
| *A. subpedatum* Ching | KUN LuJM634 | KT426985 | KT427206 | KT427075 | - |
| *A. subpedatum* Ching | KUN LuJM635 | KT426986 | KT427207 | KT427076 | - |

^#^ downloaded from NCBI Genbank.
