# Supplementary material for: Applying DNA Barcodes to Identify Closely Related Species of Ferns: A Case Study of the Chinese Adiantum (Pteridaceae)
Source: PLoS One. 2016 Sep 7;11(9):e0160611. doi: 10.1371/journal.pone.0160611 (PMC5014338; doi:10.1371/journal.pone.0160611)
Supplement: S2 Table — (DOCX) [file pone.0160611.s015.docx]

Table S2 Taxa, voucher specimens and GenBank Accession Numbers of IBR3_2 sequences.

| Taxon | - Herbarium/   Voucher number | Clone number | GenBank Accession No |
| --- | --- | --- | --- |
| *Adiantum ailaoshanense* | CSH Yan12410 | clone 7 | KX517687 |
| *A. ailaoshanense* | CSH Yan12410 | clone 8 | KX517577 |
| *A. ailaoshanense* | CSH Yan12413 | clone 1 | KX517582 |
| *A. ailaoshanense* | CSH Yan12413 | clone 2 | KX517578 |
| *A. ailaoshanense* | CSH Yan12413 | clone 3 | KX517676 |
| *A. ailaoshanense* | CSH Yan12413 | clone 4 | KX517684 |
| *A. ailaoshanense* | CSH Yan12413 | clone 5 | KX517695 |
| *A. ailaoshanense* | CSH Yan12413 | clone 6 | KX517677 |
| *A. ailaoshanense* | CSH Yan12413 | clone 9 | KX517579 |
| *A. ailaoshanense* | CSH Yan12413 | clone 10 | KX517688 |
| *A. ailaoshanense* | CSH Yan12413 | clone 11 | KX517669 |
| *A. ailaoshanense* | CSH Yan12413 | clone 12 | KX517682 |
| *A. bonatianum* | KUN LuJM216 | clone 1 | KX517737 |
| *A. bonatianum* | KUN LuJM216 | clone 2 | KX517734 |
| *A. bonatianum* | KUN LuJM216 | clone 3 | KX517700 |
| *A. bonatianum* | KUN LuJM216 | clone 5 | KX517731 |
| *A. bonatianum* | KUN LuJM216 | clone 6 | KX517741 |
| *A. bonatianum* | KUN LuJM216 | clone 7 | KX517739 |
| *A. bonatianum* | KUN LuJM216 | clone 9 | KX517740 |
| *A. bonatianum* | KUN LuJM216 | clone 10 | KX517732 |
| *A. bonatianum* | KUN LuJM216 | clone 11 | KX517704 |
| *A. bonatianum* | KUN LuJM216 | clone 12 | KX517735 |
| *A. bonatianum* | KUN LuJM438 | clone 1 | KX517701 |
| *A. bonatianum* | KUN LuJM438 | clone 2 | KX517706 |
| *A. bonatianum* | KUN LuJM438 | clone 3 | KX517733 |
| *A. bonatianum* | KUN LuJM438 | clone 4 | KX517703 |
| *A. bonatianum* | KUN LuJM438 | clone 5 | KX517702 |
| *A. bonatianum* | KUN LuJM438 | clone 6 | KX517736 |
| *A. bonatianum* | KUN LuJM438 | clone 7 | KX517705 |
| *A. bonatianum* | KUN LuJM438 | clone 8 | KX517738 |
| *A. capillus-junonis* | KUN LuJM552 * |  | KX517558 |
| *A. capillus-junonis* | KUN LuJM552 | clone 6 | KX517557 |
| *A. capillus-veneris* | KUN KUN FB412 | clone 1 | KX517753 |
| *A. capillus-veneris* | KUN KUN FB412 | clone 4 | KX517752 |
| *A. capillus-veneris* | KUN KUN FB412 | clone 6 | KX517754 |
| *A. capillus-veneris* | KUN KUN FB412 * |  | KX517755 |
| *A. caudatum* | KUN LuJM209 | clone 1 | KX517668 |
| *A. caudatum* | KUN LuJM209 | clone 3 | KX517665 |
| *A. caudatum* | KUN LuJM209 | clone 6 | KX517666 |
| *A. caudatum* | KUN LuJM209 * |  | KX517667 |
| *A. davidii* | KUN LuJM381 | clone 1 | KX517724 |
| *A. davidii* | KUN LuJM381 | clone 3 | KX517707 |
| *A. davidii* | KUN LuJM381 | clone 6 | KX517723 |
| *A. davidii var. longispium* | CSH 12692 | clone 1 | KX517726 |
| *A. davidii var. longispium* | CSH 12692 | clone 2 | KX517715 |
| *A. davidii var. longispium* | CSH 12692 | clone 3 | KX517729 |
| *A. davidii var. longispium* | CSH 12692 | clone 4 | KX517725 |
| *A. davidii var. longispium* | CSH 12692 | clone 5 | KX517728 |
| *A. davidii var. longispium* | CSH 12692 | clone 6 | KX517714 |
| *A. davidii var. longispium* | CSH 12692 | clone 7 | KX517730 |
| *A. davidii var. longispium* | CSH 12692 | clone 8 | KX517727 |
| *A. davidii var. longispium* | CSH 12692 | clone 10 | KX517716 |
| *A. davidii var. longispium* | CSH 12692 | clone 11 | KX517717 |
| *A. diaphanum* | KUN LuJM558_2 | clone 1 | KX517497 |
| *A. diaphanum* | KUN LuJM558_2 | clone 2 | KX517496 |
| *A. diaphanum* | KUN LuJM558_2 | clone 6 | KX517498 |
| *A. edgeworthii* | KUN FB229 | clone 4 | KX517653 |
| *A. edgeworthii* | KUN FB229 | clone 5 | KX517654 |
| *A. edgeworthii* | KUN FB229 | clone 8 | KX517650 |
| *A. edgeworthii* | KUN FB229 | clone 17 | KX517655 |
| *A. edgeworthii* | KUN FB229 | clone 18 | KX517646 |
| *A. edgeworthii* | KUN FB229 | clone 19 | KX517649 |
| *A. edgeworthii* | KUN FB229 | clone 20 | KX517651 |
| *A. edgeworthii* | KUN FB229 | clone 21 | KX517647 |
| *A. edgeworthii* | KUN FB229 | clone 22 | KX517652 |
| *A. edgeworthii* | KUN FB229 | clone 24 | KX517648 |
| *A. fengianum* | KUN LuJM228_2 | clone 3 | KX517699 |
| *A. fengianum* | KUN LuJM228_2 | clone 6 | KX517722 |
| *A. fimbriatum* | CSH DM5455 | clone 8 | KX517719 |
| *A. fimbriatum* | CSH DM5455 | clone 11 | KX517713 |
| *A. fimbriatum* | CSH DM5455 | clone 12 | KX517720 |
| *A. fimbriatum* | CSH DM5455 | clone 13 | KX517721 |
| *A. fimbriatum* | CSH DM5455 | clone 16 | KX517711 |
| *A. fimbriatum* | CSH DM5455 | clone 19 | KX517710 |
| *A. fimbriatum* | KUN LuJM382 | clone 3 | KX517712 |
| *A. fimbriatum* | KUN LuJM382 | clone 4 | KX517718 |
| *A. fimbriatum* | KUN LuJM382 | clone 5 | KX517708 |
| *A. fimbriatum* | KUN LuJM382 | clone 6 | KX517709 |
| *A. flabellulatum* | KUN LuJM555_1 | clone 1 | KX517758 |
| *A. flabellulatum* | KUN LuJM555_1 | clone 2 | KX517756 |
| *A. formosanum* | TAIF Kuo430 | clone 1 | KX517501 |
| *A. formosanum* | TAIF Kuo430 | clone 3 | KX517504 |
| *A. formosanum* | TAIF Kuo430 | clone 4 | KX517503 |
| *A. formosanum* | TAIF Kuo430 | clone 6 | KX517502 |
| *A. formosanum* | TAIF Kuo430* |  | KX517505 |
| *A. gravesii* | KUN LuJM451 | clone 1 | KX517638 |
| *A. gravesii* | KUN LuJM451 | clone 2 | KX517639 |
| *A. gravesii* | KUN LuJM451 * |  | KX517641 |
| *A. gravesii* | KUN LuJM163 | clone 3 | KX517644 |
| *A. gravesii* | KUN LuJM163 | clone 4 | KX517642 |
| *A. gravesii* | KUN LuJM441_1 | clone 2 | KX517645 |
| *A. gravesii* | KUN LuJM441_1 | clone 5 | KX517643 |
| *A. gravesii* | KUN LuJM451 | clone 5 | KX517640 |
| *A. hispidulum* | US Wen10243 | clone 2 | KX517494 |
| *A. hispidulum* | US Wen10243 | clone 10 | KX517492 |
| *A. hispidulum* | US Wen10243 | clone 16 | KX517493 |
| *A. hispidulum* | US Wen10771 * |  | KX517495 |
| *A. induratum* | KUN WFH008 | clone 5 | KX517757 |
| *A. juxtapositum* | CSH 13165 | clone 3 | KX517611 |
| *A. juxtapositum* | CSH 13165 | clone 5 | KX517612 |
| *A. juxtapositum* | CSH 13165 | clone 6 | KX517617 |
| *A. juxtapositum* | CSH 13165 | clone 7 | KX517609 |
| *A. juxtapositum* | CSH 13165 | clone 8 | KX517616 |
| *A. juxtapositum* | CSH 13165 | clone 10 | KX517618 |
| *A. juxtapositum* | CSH 13165 | clone 12 | KX517620 |
| *A. juxtapositum* | KUN WFH060 | clone 1 | KX517614 |
| *A. juxtapositum* | KUN WFH060 | clone 2 | KX517619 |
| *A. juxtapositum* | KUN WFH060 | clone 3 | KX517608 |
| *A. juxtapositum* | KUN WFH060 | clone 4 | KX517607 |
| *A. juxtapositum* | KUN WFH060 | clone 5 | KX517613 |
| *A. juxtapositum* | KUN WFH061 | clone 4 | KX517610 |
| *A. juxtapositum* | KUN WFH061 | clone 5 | KX517621 |
| *A. juxtapositum* | KUN WFH061 | clone 7 | KX517606 |
| *A. juxtapositum* | KUN WFH061 | clone 8 | KX517603 |
| *A. juxtapositum* | KUN WFH061 | clone 10 | KX517604 |
| *A. juxtapositum* | KUN WFH061 | clone 11 | KX517615 |
| *A. juxtapositum* | KUN WFH061 | clone 12 | KX517605 |
| *A. lianxianense* | KUN LuJM120 | clone 1 | KX517601 |
| *A. lianxianense* | KUN LuJM120 | clone 2 | KX517602 |
| *A. lianxianense* | KUN LuJM120 | clone 4 | KX517623 |
| *A. lianxianense* | KUN LuJM120 | clone 5 | KX517624 |
| *A. lianxianense* | KUN LuJM120 | clone 6 | KX517622 |
| *A. malesianum* | KUN FB260 | clone 1 | KX517659 |
| *A. malesianum* | KUN FB260 | clone 3 | KX517660 |
| *A. malesianum* | KUN WFH049 | clone 4 | KX517664 |
| *A. malesianum* | KUN WFH049 | clone 5 | KX517661 |
| *A. mariesii* | CSH ZXL09685 | clone 6 | KX517633 |
| *A. mariesii* | CSH ZXL09685 | clone 9 | KX517634 |
| *A. mariesii* | CSH ZXL09685 | clone 12 | KX517635 |
| *A. mariesii* | CSH ZXL09685 | clone 13 | KX517636 |
| *A. mariesii* | CSH ZXL09685 | clone 14 | KX517637 |
| *A. mariesii* | CSH ZXL09685 | clone 15 | KX517632 |
| *A. mariesii* | CSH ZXL09685 | clone 17 | KX517630 |
| *A. mariesii* | CSH ZXL09685 | clone 18 | KX517631 |
| *A. mariesii* | CSH ZXL09685 | clone 22 | KX517629 |
| *A. meishanianum* | KUN WFH043 | clone 1 | KX517656 |
| *A. meishanianum* | KUN WFH043 | clone 2 | KX517568 |
| *A. meishanianum* | KUN WFH043 | clone 3 | KX517574 |
| *A. meishanianum* | KUN WFH043 | clone 6 | KX517599 |
| *A. meishanianum* | KUN WFH043 | clone 7 | KX517658 |
| *A. meishanianum* | KUN WFH043 | clone 8 | KX517583 |
| *A. meishanianum* | TAIF Kuo n.s. | clone 2 | KX517657 |
| *A. meishanianum* | TAIF Kuo n.s. | clone 3 | KX517663 |
| *A. meishanianum* | TAIF Kuo n.s. | clone 9 | KX517662 |
| *A. menglianense* | KUN LuJM114 | clone 4 | KX517589 |
| *A. menglianense* | KUN LuJM114 | clone 8 | KX517581 |
| *A. menglianense* | KUN LuJM114 | clone 9 | KX517584 |
| *A. menglianense* | KUN LuJM114 | clone 13 | KX517586 |
| *A. menglianense* | KUN LuJM114 | clone 14 | KX517591 |
| *A. menglianense* | KUN LuJM114 | clone 15 | KX517566 |
| *A. menglianense* | KUN LuJM114 | clone 17 | KX517580 |
| *A. menglianense* | KUN LuJM114 | clone 18 | KX517585 |
| *A. menglianense* | KUN WFH017 | clone 1 | KX517569 |
| *A. menglianense* | KUN WFH017 | clone 3 | KX517590 |
| *A. menglianense* | KUN WFH017 | clone 4 | KX517592 |
| *A. menglianense* | KUN WFH017 | clone 5 | KX517594 |
| *A. menglianense* | KUN WFH017 | clone 10 | KX517571 |
| *A. menglianense* | KUN WFH017 | clone 11 | KX517567 |
| *A. menglianense* | KUN WFH017 | clone 15 | KX517575 |
| *A. menglianense* | KUN WFH017 | clone 16 | KX517593 |
| *A. menglianense* | KUN WFH017 | clone 20 | KX517576 |
| *A. menglianense* | KUN WFH017 * |  | KX517595 |
| *A. menglianense* | KUN WFH033 * |  | KX517570 |
| *A. menglianense* | KUN WFH039 | clone 1 | KX517588 |
| *A. menglianense* | KUN WFH039 | clone 2 | KX517572 |
| *A. menglianense* | KUN WFH039 | clone 3 | KX517587 |
| *A. menglianense* | KUN WFH039 | clone 8 | KX517596 |
| *A. menglianense* | KUN WFH039 | clone 9 | KX517573 |
| *A. menglianense* | KUN WFH039 | clone 10 | KX517597 |
| *A. menglianense* | KUN WFH039 | clone 11 | KX517598 |
| *A. menglianense* | KUN WFH039 * |  | KX517600 |
| *A. menglianense* | KUN WFH057 * |  | KX517565 |
| *A. monochlamys* | TAIF Kuo n.s. | clone 17 | KX517742 |
| *A. myriosorum* | KUN LuJM480 | clone 1 | KX517523 |
| *A. myriosorum* | KUN LuJM480 | clone 4 | KX517522 |
| *A. myriosorum* | KUN LuJM480 | clone 5 | KX517520 |
| *A. myriosorum* | KUN LuJM480 | clone 6 | KX517521 |
| *A. myriosorum* | HSAT PJY4_2 * |  | KX517524 |
| *A. pedatum* | KUN LuJM342 | clone 3 | KX517526 |
| *A. pedatum* | KUN LuJM342 | clone 8 | KX517525 |
| *A. philippense* | KUN FB270 * |  | KX517563 |
| *A. philippense* | KUN FB336 | clone 2 | KX517561 |
| *A. philippense* | KUN FB336 | clone 3 | KX517562 |
| *A. philippense* | KUN FB336 * |  | KX517564 |
| *A. philippense* | KUN FB534 * |  | KX517560 |
| *A. philippense* | KUN WFH036 * |  | KX517559 |
| *A. refractum* | KUN LuJM222* |  | KX517512 |
| *A. refractum* | KUN LuJM231 | clone 1 | KX517506 |
| *A. refractum* | KUN LuJM231 | clone 3 | KX517507 |
| *A. refractum* | KUN LuJM231 * |  | KX517510 |
| *A. refractum* | KUN LuJM256 | clone 2 | KX517499 |
| *A. refractum* | KUN LuJM256 | clone 4 | KX517500 |
| *A. refractum* | KUN LuJM256 | clone 5 | KX517508 |
| *A. refractum* | KUN LuJM256 | clone 6 | KX517509 |
| *A. refractum* | KUN LuJM256 * |  | KX517511 |
| *A. reniforme* | IBSC T-PH002 | clone 1 | KX517532 |
| *A. reniforme* | IBSC T-PH002 | clone 2 | KX517533 |
| *A. reniforme* | IBSC T-PH002 | clone 4 | KX517535 |
| *A. reniforme* | IBSC Xing & Wang 011 | clone 1 | KX517534 |
| *A. reniforme* | IBSC Xing & Wang 011 | clone 2 | KX517553 |
| *A. reniforme* | IBSC Xing & Wang 011 | clone 3 | KX517531 |
| *A. reniforme* | IBSC Xing & Wang 011 | clone 4 | KX517527 |
| *A. reniforme* | IBSC Xing & Wang 011 | clone 5 | KX517552 |
| *A. reniforme* | IBSC Xing & Wang 011 | clone 7 | KX517529 |
| *A. reniforme* | IBSC Xing & Wang 011 | clone 8 | KX517528 |
| *A. reniforme* | IBSC Xing & Wang 011 | clone 9 | KX517551 |
| *A. reniforme* | IBSC Xing & Wang 011 | clone 10 | KX517530 |
| *A. reniforme* | IBSC Xing & Wang 011 | clone 12 | KX517550 |
| *A. reniforme var. sinense* | IBSC Wah001 | clone 2 | KX517544 |
| *A. reniforme var. sinense* | IBSC Wah001 | clone 3 | KX517545 |
| *A. reniforme var. sinense* | IBSC Wah001 | clone 4 | KX517540 |
| *A. reniforme var. sinense* | IBSC Wah001 | clone 5 | KX517547 |
| *A. reniforme var. sinense* | IBSC Wah001 | clone 6 | KX517538 |
| *A. reniforme var. sinense* | IBSC Wah001 | clone 9 | KX517549 |
| *A. reniforme var. sinense* | IBSC Wah001 | clone 12 | KX517536 |
| *A. reniforme var. sinense* | IBSC Wah005 | clone 1 | KX517543 |
| *A. reniforme var. sinense* | IBSC Wah005 | clone 6 | KX517546 |
| *A. reniforme var. sinense* | IBSC Wah005 | clone 7 | KX517539 |
| *A. reniforme var. sinense* | IBSC Wah005 | clone 8 | KX517542 |
| *A. reniforme var. sinense* | IBSC Wah005 | clone 10 | KX517548 |
| *A. reniforme var. sinense* | IBSC Wah005 | clone 11 | KX517537 |
| *A. reniforme var. sinense* | KUN LuJM238_2 * |  | KX517541 |
| *A. roborowskii var. roborowskii* | KUN LuJM280 | clone 5 | KX517749 |
| *A. roborowskii var. roborowskii* | KUN LuJM280 | clone 20 | KX517743 |
| *A. roborowskii var. roborowskii* | KUN LuJM280 | clone 9 | KX517698 |
| *A. roborowskii var. roborowskii* | KUN LuJM280 | clone 18 | KX517751 |
| *A. roborowskii var. roborowskii* | KUN LuJM280 | clone 13 | KX517745 |
| *A. roborowskii var. roborowskii* | KUN LuJM280 | clone 8 | KX517747 |
| *A.roborowskii var. robustum* | BJFC HB05 | clone 4 | KX517744 |
| *A. roborowskii var. taiwanianum* | TAIF Wade909 | clone 19 | KX517746 |
| *A. roborowskii var. taiwanianum* | TAIF Wade909 | clone 5 | KX517748 |
| *A. roborowskii var. taiwanianum* | TAIF Wade909 | clone 11 | KX517750 |
| *A. sinicum* | KUN FB264* |  | KX517686 |
| *A. sinicum* | KUN FB870 | clone 1 | KX517678 |
| *A. sinicum* | KUN FB870 | clone 2 | KX517674 |
| *A. sinicum* | KUN FB870 | clone 3 | KX517670 |
| *A. sinicum* | KUN FB870 | clone 4 | KX517685 |
| *A. sinicum* | KUN FB870 | clone 5 | KX517697 |
| *A. sinicum* | KUN FB871 | clone 2 | KX517681 |
| *A. sinicum* | KUN FB871 | clone 3 | KX517675 |
| *A. sinicum* | KUN FB871 | clone 5 | KX517679 |
| *A. sinicum* | KUN FB871 | clone 6 | KX517696 |
| *A. sinicum* | KUN FB871 | clone 7 | KX517680 |
| *A. sinicum* | KUN FB871 | clone 8 | KX517671 |
| *A. sinicum* | KUN FB871 | clone 11 | KX517683 |
| *A. sinicum* | KUN FB871 | clone 12 | KX517689 |
| *A. sinicum* | KUN FB872 | clone 1 | KX517690 |
| *A. sinicum* | KUN FB872 | clone 2 | KX517693 |
| *A. sinicum* | KUN FB872 | clone 4 | KX517691 |
| *A. sinicum* | KUN FB872 | clone 5 | KX517694 |
| *A. sinicum* | KUN FB872 | clone 6 | KX517692 |
| *A. sinicum* | KUN LuJM269* |  | KX517672 |
| *A. sinicum* | KUN WFH014* |  | KX517673 |
| *A. soboliferum* | KUN WFH038 | clone 10 | KX517556 |
| *A. soboliferum* | KUN SunH17076 | clone 11 | KX517554 |
| *A. soboliferum* | KUN SunH17076 | clone 4 | KX517555 |
| *A. sp.* | KUN CPC050 | clone 4 | KX517625 |
| *A. sp.* | KUN CPC050 | clone 6 | KX517626 |
| *A. sp.* | KUN CPC050 | clone 9 | KX517628 |
| *A. sp.* | KUN CPC050* |  | KX517627 |
| *A. subpedatum* | KUN LuJM635_3 | clone 1 | KX517514 |
| *A. subpedatum* | KUN LuJM635_3 | clone 2 | KX517515 |
| *A. subpedatum* | KUN LuJM635_3 | clone 3 | KX517513 |
| *A. subpedatum* | KUN LuJM635_3 | clone 6 | KX517516 |
| *A. subpedatum* | KUN LuJM635_3 | clone 8 | KX517517 |
| *A. subpedatum* | KUN LuJM635_3 | clone 10 | KX517519 |
| *A. subpedatum* | KUN LuJM635_3 | clone 12 | KX517518 |

* The sequences via direct sequencing
