# Supplementary material for: Applying DNA Barcodes to Identify Closely Related Species of Ferns: A Case Study of the Chinese Adiantum (Pteridaceae)
Source: PLoS One. 2016 Sep 7;11(9):e0160611. doi: 10.1371/journal.pone.0160611 (PMC5014338; doi:10.1371/journal.pone.0160611)
Supplement: S3 Table — (DOCX) [file pone.0160611.s016.docx]

Table S3 Primers used in this study.

| DNA regions | Primer pairs | Primer sequences(5'—3') | Thermocycling conditions | Reference |
| --- | --- | --- | --- | --- |
| *rbc*L | 1F | ATG TCA CCA CAA ACA GAG ACT AAA GC | 95 ℃, 3 min; [35 cycles: 95 ℃,1 min; 51 ℃,1 min; 72℃, 80 s ]; 72 ℃, 10 min | Little & Barrington 2003[86] |
|  | 1379R | TCACAAGCAGCAGCTAGTTCAGGACTC |  | Little & Barrington 2003[86] |
| *trn*H*-psb*A | trnH | CGC GCA TGG TGG ATT CAC AAT CC | 94 ℃, 2 min; [35 cycles: 94 ℃, 35 s; 52 ℃, 40 s; 72 ℃, 45 s ]; 72 ℃, 7 min | Tate & Simpson 2003[87] |
|  | psbA | GTT ATG CAT GAA CGT AAT GCT C |  | Sang *et al.* 1997[88] |
| *rpS4-trn*S | rps4.5 | TAC CGA GGG TTC GAA TC | 95 ℃, 3 min; [35 cycles: 94 ℃,30 s; 58 ℃, 45 s; 72 ℃, 80 s ]; 72 ℃, 10 min | Souza-Chies *et al.* 1997[89] |
|  | trnS | TTA CCG AGG GTT CGA ATC CCT C |  | Shaw *et al.* 2005[90] |
| *trn*L-F | c | CGAAATCGGTAGACGCTACG | 95 ℃, 3 min; [35 cycles: 95 ℃,1 min; 51 ℃,1 min; 72 ℃, 80 s ]; 72 ℃, 10 min | Taberlet *et al.* 1991[91] |
|  | f | ATITGAACTGGTGACACGAG |  | Taberlet *et al.* 1991[91] |
| *mat*K | FWPtmatKF1 | AYGAAAGYCRYTTAYGGATCT | 95 ℃, 5 min; [35 cycles: 94 ℃,1 min; 53 ℃,1 min; 72 ℃, 2 min]; 72 ℃, 10 min | Li *et al*. 2011[12] |
|  | FWPtmatK rAGK | CGTATTGTACTYCTATGTTTRCCAGC |  | Li *et al.*2011[12] |
| *gap*C*p* | ESGAPCP8F1 | ATYCCAAGYTCAACTGGTGCTGC | 94 ℃, 5 min; [35 cycles: 94 ℃,1 min; 55 ℃,1 min; 72 ℃, 2 min]; 72 ℃, 10 min | Schuettpelz *et al.* 2008[41] |
|  | ESGAPCP11R1 | GTATCCCCAYTCRTTGTCRTACC |  | Schuettpelz *et al.*2008[41] |
| *pgi*C | 14F | GTGCTTCTGGGTCTTTTGAGTG | 95 ℃, 3 min; [3 cycles: 94 ℃,1 min; 56 ℃,1 min; 72 ℃, 2 min]; [3 cycles: 94 ℃,1 min; 53 ℃,1 min; 72 ℃, 2 min];[34 cycles: 94 ℃,45 s; 50 ℃,45 s; 72 ℃, 90 s];72 ℃, 8 min | Ishikawa *et al.* 2002[40] |
|  | 15F | TGTTGAATGTGAGGAGCAAA |  | Ishikawa *et al.* 2002[40] |
|  | 16R | GTTGTCCATTAGTTCCAGGTTCCCC |  | Ishikawa *et al.* 2002[40] |
| LEAFY | leafyF | TGA AGT GCA GCA GAT GTC AA | 94 ℃, 2 min; [35 cycles: 94 ℃,1 min; 57 ℃,1 min; 72 ℃, 1 min]; 72 ℃, 5 min | Shepherd *et al.* 2008[42] |
|  | leafyR | GCT AGC ACC TTT CAG CTT GG |  | Shepherd *et al.* 2008[42] |
| ITS | ITS4 | TCCTCCGCTTATTGATATGC | 94 ℃, 2 min; [35 cycles: 94 ℃,40 s; 55℃,45 s; 72 ℃, 55 s]; 72 ℃, 7 min | White *et al.* 1990[92] |
|  | ITS5 | GGAAGGTAAAAGTCAAGG |  | White *et al.*1990[92] |
| ITS2 | GYM_5.8S F2 | GYAGAATCCCGTGARTCATC | 94 ℃, 2 min; [35 cycles: 94 ℃,35 s; 52℃,40 s; 72 ℃, 50 s]; 72 ℃, 7 min | Gao *et al.* 2012[93] |
|  | ITS4 | TCCTCCGCTTATTGATATGC |  | White *et al.*1990[92] |
| DET1 | det1-335all | TATGAYGTGGARTGCCCAGAT | 94 ℃, 3 min; [35 cycles: 95 ℃,1 min; 55℃,1 min; 72 ℃, 1 min]; 72 ℃, 7 min | Rothfels *et al.* 2013[48] |
|  | det1-906all | TCTCTGCAGAAHKGYCCAA |  | Rothfels *et al*. 2013[48] |
| IBR3_2 | 4321F5 | ATGACYGAACCAGATGTKGCDTCVTCRGATGC | 95 ℃, 3 min; [35 cycles: 95 ℃,1 min; 65℃,2 min; 72 ℃, 1 min]; 72 ℃, 7 min | Rothfels *et al*. 2013[48] |
|  | 4321R6 | TGRTGGAGYCTKCCTGGGCCTA |  | Rothfels *et al*. 2013[48] |
| SQD1_1 | EMSQD1E1F6 | GCAAGGGTACHAAGGTHATGATCATAGG | 95 ℃,3 min; [35 cycles: 95 ℃,1 min; 55℃,2 min; 72 ℃, 1 min]; 72 ℃, 7 min | Rothfels *et al*. 2013[48] |
|  | EMSQD1E1R2 | CCTTTDCCRTARACTGTAAGAGGATG |  | Rothfels *et al*. 2013[48] |

86. Little DP, Barrington DS. Major evolutionary events in the origin and diversification of the fern genus *Polystichum* (Dryopteridaceae). Am J Bot. 2003; 90: 508–514.

87. Tate JA, Simpson BB. Paraphyly of *Tarasa* (Malvaceae) and diverse origins of the polyploid species. Syst Bot. 2003; 28: 723–737.

88. Sang T, Crawford D, Stuessy T. Chloroplast DNA phylogeny, reticulate evolution, and biogeography of *Paeonia* (Paeoniaceae). Am J Bot. 1997; 84: 1120–1136.

89. Souza-Chies TT, Bittar G, Nadot S, Carter L, Besin E, Lejeune B. Phylogenetic analysis of Iridaceae with parsimony and distance methods using the plastid gene *rps4*. Plant Syst Evol. 1997; 204: 109–123.

90. Shaw J, Lickey EB, Beck JT, Farmer SB, Liu W, Miller J, et al. The tortoise and the hare II: relative utility of 21 noncoding chloroplast DNA sequences for phylogenetic analysis. Am J Bot. 2005;92: 142–166.

91. Taberlet P, Gielly L, Pautou G, Bouvet J. Universal primers for amplification of three non-coding regions of chloroplast DNA. Plant Mol Biol. 1991;17: 1105–1109.

92. White TJ, Bruns T, Lee S, Taylor J. Amplification and direct sequencing of fungal ribosomal RNA genes for phylogenetics. In: Innis MA, Gelfand DH, Sninsky JJ, White TJ, editors. PCR Protocols: A Guide to Methods and Applications. San Diego, California: Academic Press; 1990. pp. 315–322.

93. Gao LM, Liu J, Cai J, **Yang JB, Zhang T, Li DZ***.* A synopsis of technical notes on the standards for plant DNA barcoding. Plant Diversity and Resources. 2012; 34: 592–606.
