# Supplementary material for: Applying DNA Barcodes to Identify Closely Related Species of Ferns: A Case Study of the Chinese Adiantum (Pteridaceae)
Source: PLoS One. 2016 Sep 7;11(9):e0160611. doi: 10.1371/journal.pone.0160611 (PMC5014338; doi:10.1371/journal.pone.0160611)
Supplement: S1 Fig — (PDF) [file pone.0160611.s001.pdf]

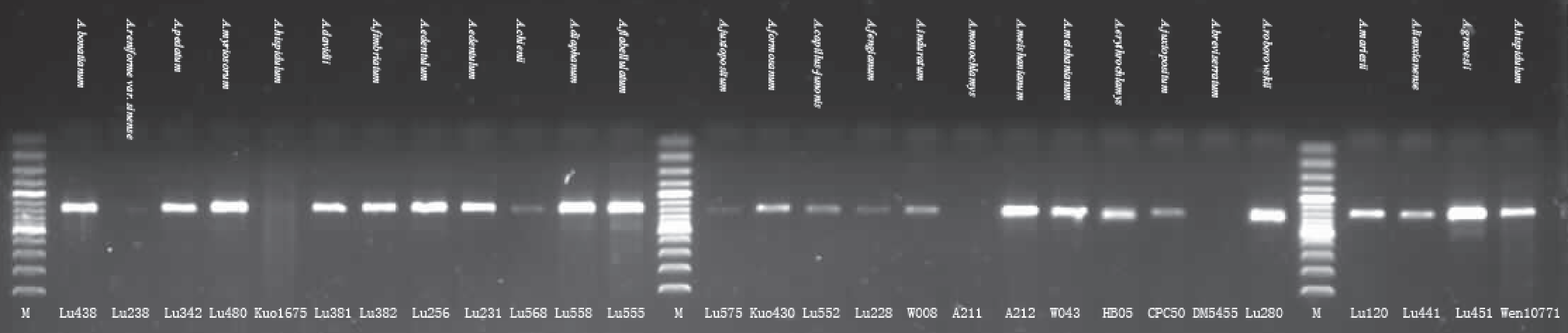

DNA region: IBR3_2. Primer pairs: 4321F5,4321R6. PCR protocol: 95℃，3min, 35×{95℃，1min；65℃，2min；72℃，1min} 72℃，7min
